# Mesozooplankton distribution in the Guinea Current Large Marine Ecosystem (GCLME) during the west African monsoon: a comparison across subsystems

**DOI:** 10.1093/plankt/fbag050

**Published:** 2026-07-02

**Authors:** Olga Anadoli, Hawa Bint Yaqub, Maryse Aka, Estelle S Konan, Emmanuel Acheampong, Jerome Adonis Zuweh, Lynette John, Stamatina Isari

**Affiliations:** Institute of Marine Research, Plankton Research Group, Nordnesgaten 50, Bergen 5005, Vestland, Norway; Fisheries Commission, Marine Fisheries Management, GA-079-5564 Egypt Lane, Ridge, P.O. Box GP 630, Accra, Ghana; Oceanological Research Center, Environment Department, 1071 Rue Roger Zinsou, BPV 18 Abidjan, Côte d'Ivoire; Oceanological Research Center, Environment Department, 1071 Rue Roger Zinsou, BPV 18 Abidjan, Côte d'Ivoire; Department of Fisheries and Aquatic Sciences, Africa Centre of Excellence in Coastal Resilience, UCC Old Site, University of Cape Coast, Central Region, CC-146-9497, Cape Coast, Ghana; National Fisheries and Aquaculture Authority, Adjacent LPRC, Bushrod Island, P.O. Box 1384 1000 Monrovia, Republic of Liberia; Institute of Marine Biology and Oceanography, Fourah Bay College, University of Sierra Leone, Mount Aureol, Freetown, Sierra Leone; Institute of Marine Research, Plankton Research Group, Nordnesgaten 50, Bergen 5005, Vestland, Norway

**Keywords:** mesozooplankton distribution, copepods, assemblage structure, Gulf of Guinea, African monsoon, upwelling

## Abstract

The Guinea Current Large Marine Ecosystem (GCLME), located in the Eastern Equatorial Atlantic, is highly productive and supports important coastal livelihoods and fisheries across West Africa. Mesozooplankton and hydrographic sampling were conducted across an extended area of the GCLME, from Guinea-Bissau to Ghana, during two consecutive surveys between July and September 2017. The sampling coincided with the West African Monsoon and the major upwelling period in the central GCLME (C-GCLME: Côte d’Ivoire, Ghana), while the western GCLME (W-GCLME: Guinea-Bissau to Liberia) experienced its main rainy season. Contrasting hydrographic regimes between the two subsystems were reflected in the zooplankton community structure and distribution patterns. The cooler upwelled waters of the C-GCLME supported significantly higher mesozooplankton stock and a copepod assemblage characterized by opportunistic, or upwelling-associated species (i.e. *Calanoides natalis, Centropages chierchiae*), most prominently along the Ivorian and eastern Ghanaian coasts. In contrast, the W-GCLME was characterized by thermally stratified, low-salinity waters influenced by riverine inputs, supporting thermophilic, as well as opportunistic species, particularly along the Guinea–Sierra Leone Plateau. These results highlight clear ecological differences between the two subsystems and provide baseline information on zooplankton biomass and biodiversity, relevant to local food web dynamics and ecosystem-based management efforts.

## INTRODUCTION

The Guinea Current Large Marine Ecosystem (GCLME), located along the equatorial West African coast, provides vital ecosystem services that sustain millions of livelihoods (Binet and Marchal, 1993; Satia, 2016). It spans approximately from Guinea–Bissau to Gabon, encompassing contrasting subsystems (Binet and Marchal, 1993; Hardman-Mountford and McGlade, 2002a, 2002b), characterized by high biological productivity that supports coastal fisheries essential to food security in the region (Binet and Marchal, 1993). Subsystem productivity levels are strongly influenced by topographical features, hydrographic structures, environmental forcing and seasonally driven upwelling events, which vary across the entire region in both nature and timing (Bakun, 1978; Binet and Marchal, 1993; Hardman-Mountford and McGlade, 2002b; Nieto and Mélin, 2017).

The western part of the basin - western GCLME (W-GCLME), extending from Guinea-Bissau to Liberia, features the largest continental shelf in West Africa. Its northern section is influenced by the Mauritanian-Senegalese seasonal upwelling (Vázquez *et al.*, 2023). As a result, the coasts of Guinea-Bissau and Guinea experience upwelling processes during the boreal winter under the influence of the Canary Current (Nieto and Mélin, 2017); during the summer, wind favors downwelling which leads to pronounced decrease in chlorophyll concentrations in the surface layers of the area (Vázquez *et al*., 2023). On the other hand, the central GCLME (C-GCLME), particularly the Ivorian/Ghanaian region, forms part of the central West African upwelling system (Hardman-Mountford and McGlade, 2002a). Seasonal upwelling along this zonal coastline is driven by wind and current dynamics and modulated by pressure variations in the tropical Atlantic (Djakouré *et al*., 2017; Djakouré *et al.*, 2024a). A major upwelling period in this region occurs from July to September, coinciding with the West African Monsoon and the northward shift of the Intertropical Convergence Zone (ITCZ) (Sultan *et al*., 2007; Djakouré *et al*., 2024b). This shift marks the rainy season in the northern W-GCLME and the peaks in river discharge (Gu and Adler, 2004; Efon *et al*., 2023), while C-GCLME experiences a minor dry season (Djakouré *et al*., 2024b).

The major upwelling season is recognized as the most productive period in C-GCLME, with large increases in primary (Dandonneau, 1973; Binet, 1983a; Anang, 2000; John *et al*., 2002) and secondary productivity (Binet, 1983b; Wiafe *et al*., 2008, 2016). This seasonal productivity bloom is driven by nutrient enrichment associated with coastal upwelling, which ultimately supports the high fish biomass and sustains populations of small pelagic fish, such as sardinellas, in the region (Binet, 1995; Pezennec and Koranteng, 1998). Assessing the current status of zooplankton communities is therefore particularly important as zooplankton community structure together with their biological and ecophysiological traits, influence key ecosystem processes such as energy transfer pathways and biochemical cycling, potentially, triggering abrupt and irreversible changes in marine ecosystem services (Ratnarajah *et al*., 2023; Grigoratou *et al*., 2025).

To investigate how interannual fluctuations in zooplankton abundance and biomass relate to fishery yields, long-term zooplankton monitoring programs closely tied to fisheries management were initiated in the late 1960s along the coasts of Côte d’Ivoire and Ghana. A prominent example of these efforts was a decade-long monitoring program (1969–1979) at a coastal station near Abidjan, (Côte d’Ivoire), which, together with several shelf-wide surveys, laid the groundwork for understanding seasonal and interannual variations of mesozooplankton stock in relation to key environmental drivers (Binet, 1976; Le Borgne and Binet, 1979). These efforts contributed to baseline knowledge on the copepod spatiotemporal distribution (Binet, 1978) and their life cycles (Binet, 1977a), as well as the seasonal variation and vertical distribution of other major zooplankton groups (Binet, 1977b).

Another notable example of zooplankton time series within the region occurred along an oceanographic transect off the coast of Ghana (i.e. Tema), over a period of 24 years (1969–1992). This work provided valuable insights into impacts associated with sea surface warming (climate change), reporting a significant downward trend in zooplankton biomass, particularly during the major upwelling season (Mensah, 1995; Yaqub, 2000; Wiafe *et al*., 2008). Despite these findings and other environmental challenges confronting C-GCLME, the most recent assessments of zooplankton assemblage structure in the region date back to the mid-1990s (Wiafe *et al*., 2016). Since then, all routine monitoring efforts have ceased across the region, leading to a critical gap in long-term ecosystem observation. This lack of sustained effort is particularly acute in the W-GCLME. This area is severely under sampled and lacks any established monitoring program, aside from a few small-scale studies carried out at limited spatial resolution (Binet, 1983b; Ndour *et al*., 2018). The W-GCLME can therefore be described as a “blind spot” that needs to be addressed in the plankton records to enable science-based management of the marine ecosystem along the West African coast.

In light of the observed impacts of global warming on zooplankton biomass in the C-GCLME (Wiafe *et al.*, 2008), as well as emerging evidence of potential synergistic effects of climate change and pollution on zooplankton ecophysiology in this hotspot of anthropogenic stressors (Hernández Ruiz *et al.*, 2021; Albani *et al.*, 2023), there is growing need to assess the current status of zooplankton communities at a regional level. Here, we present data collected across a large part of the GCLME system within the framework of the EAF-Nansen Programme of the Food and Agriculture Organization of the United Nations (FAO). Mesozooplankton and hydrographic data were collected in 2017, between July and September, a period that coincides with the West African monsoon season (Citeau *et al.*, 1989) and major upwelling in the C-GCLME (Binet and Marchal, 1993). In contrast to the overall low productivity of W-GCLME (Binet, 1983b), we expect that upwelling conditions in the C-GCLME would determine the structure of mesozooplankton community, leading to a seasonally high productive environment as indicated by monitoring efforts several decades ago. Additionally, mesoscale variability of the hydrological features and riverine input were anticipated to contribute to further heterogeneity within and between subsystems. This study provides essential baseline information on mesozooplankton abundance and biomass, distribution patterns, community structure and diversity, serving as a critical reference point for future research at detecting ecological and environmental shifts. These insights are not only relevant for regional fisheries management but also crucial for advancing our knowledge and understanding of climate change dynamics, particularly through its effects on zooplankton, which can respond rapidly to environmental fluctuations.

## METHODS

### Study area

**Fig. 1:**
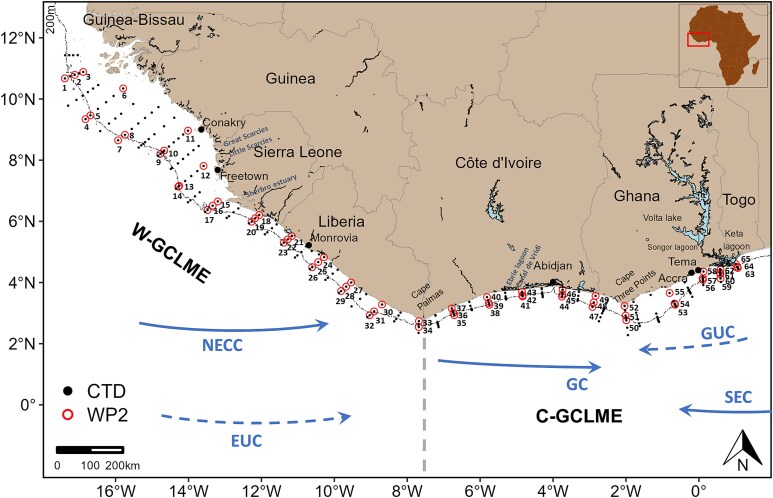
Map showing the topography and sampling grid of the W-GCLME and C-GCLME. Locations of hydrographic (CTD) and mesozooplankton (WP2) sampling stations are indicated. Current circulation patterns are based on Binet and Marchal (1993), including north equatorial counter current (NECC), Guinea current (GC), south equatorial current (SEC), Guinea under current (GUC), and equatorial under current (EUC). Major surface currents are shown with solid lines, while subsurface currents are depicted with dashed lines. The dashed gray line indicates the boundary between the W-GCLME and C-GCLME regions as defined in this work.

The study covered the Exclusive Economic Zones of six GCLME countries, structured into two subsystems: C-GCLME (Côte d’Ivoire, Ghana) and W-GCLME (Guinea-Bissau, Guinea, Sierra Leone, Liberia) (Fig. 1). Both subsystems are influenced by the warm waters of the tropical Atlantic; they also exhibit seasonal cold features associated with upwelling events that show high interannual variability both in terms of intensity and spatial extent (Hardman-Mountford and McGlade, 2002b). The W-GCLME is generally characterized by permanently warm, low-salinity waters and substantial riverine input (Binet, 1983b; Hardman-Mountford and McGlade, 2002b). The influence of the Senegalese-Mauritanian coastal upwelling becomes evident in this area from December to March or April; the movement of the water during this period is mostly southward displacing the tropical surface waters (Hardman-Mountford and McGlade, 2002b). The waters shift northward again from June to September, following the seasonal migration of the ITCZ (Hardman-Mountford and McGlade, 2002b).

On the other hand, C-GCLME experiences two upwelling seasons per year with varying intensity: a major upwelling from July to September and a minor one from January to March (Roy, 1995). The major upwelling, that propagates westward from along the coast, from Ghana to Côte d’Ivoire, has been considered influenced by multiple factors (Roy, 1995), including the shallow (0–25 m), eastward-flowing Guinea Current fed by the North Equatorial Counter-Current (Fig. 1), key topographic features such as Cape Palmas and Cape Three Points, and remote forcing mechanisms like coastally trapped waves. The upwelling events start from the east of Cape Three Points in Ghana, where it has the greatest strength, and shift westwards toward Cape Palmas on the Coast of Liberia (Hardman-Mountford and McGlade, 2002b).

Although the upwelling events in the GCLME are considered as the largest marine enrichment mechanism on the coast of West Africa (Binet and Marchal, 1993), considerable nutrient input from land drainage and river deltas also occur within the zone. Within the C-GCLME for example, the Volta estuary may serve as major source of nutrients before the commencement of the upwelling in late June (Binet and Marchal, 1993). In the W-GCLME, the Guinea-Sierra Leone Plateau receives a substantial input of fresh water during the African Monsoon from complex estuarine systems (e.g. estuaries in the vicinity of Freetown: Great Scarcies, Little Scarcies, Sherbro River) (Efon *et al*., 2023).

### Sampling design

The study area was covered during two oceanographic surveys conducted by the R/V Dr Fridtjof Nansen. The surveys were done in succession; first within the W-GCLME between 21 July and 20 August 2017, followed by the C-GCLME between 22 August and 13 September 2017.

Mesozooplankton sampling grid included a total of 22 transects (W-GCLME: 12, C-GCLME: 10) positioned perpendicular to the coast (Fig. 1). Samples were collected at 65 stations (W-GCLME: 34; C-GCLME: 31), located at three isobaths (i.e. ~ 30, 100, 500 m) within these transects. A WP2 net (56 cm diameter, 180 μm mesh size), equipped with a mechanical flowmeter, was towed vertically at a speed of ~ 0.5 ms^−1^. Sampling was conducted from the depth of 200 m to the surface (or from 3 m above the sea floor at shallower stations) during both day or night (Fig. S1). Although light conditions are not expected to have influenced the majority of stations (i.e. < 200 m bottom depth) as the entire water column was sampled, mesozooplankton diel vertical migration (DVM) could have affected the deeper stations (≥500 m depth).

Vertical profiles of hydrographic parameters—temperature, salinity and fluorescence—were taken using a Seabird 911 conductivity, temperature and depth (CTD) probe across a denser grid, comprising a total of 245 stations, including the plankton sampling locations (Fig. 1, W-GCLME: 120 stations, C-GCLME: 125 stations). The CTD was equipped with a sensor for fluorescence (Chelsea Mk III Aquatracka fluorometer) that was used to estimate chlorophyll-*α* (Chl-a) concentrations (mg m^−3^). Fluorescence values were validated against Chl-a measurements in water samples collected at predefined depths using Niskin bottles mounted on a rosette system attached to the CTD. Salinity measurements were cross verified onboard using a Portasal Salinometer (Model 8410A).

### Sample analysis

After collection, all WP2 samples were halved with a Motoda plankton splitter. The first half was collected on pre-weighed aluminum trays and dried in the oven at ~ 60°C for 24 h following the recommendation of Postel *et al.* (2000) for zooplankton dry weight determination (proxy of biomass). Samples were weighted using a Sartorius Quintix 224-1CEU analytical balance (precision: 0.1 mg). The second half was preserved in 4% sea water formaldehyde solution buffered with borax to be used for taxonomic identification. Formalin preserved samples from three stations (26, 29 and 32) were not available for abundance estimation.

Mesozooplankton subsamples representing up to 20% of the preserved sample were taken using a 5 mL Hensen-Stempel pipette and examined under a stereomicroscope for the estimation of taxonomic composition and abundance. The organisms were identified to major zooplankton groups, while copepods and diplostracans were, in most cases, identified to species level. The taxonomic identification was based on several sources (Rose, 1933; Trégouboff and Rose, 1957; Boltovskoy *et al*., 1999; Wiafe and Frid, 2001; Boxshall and Halsey, 2004; Richardson *et al*., 2013; Mazzocchi, 2020; Khelifi-Touhami and Ounissi, 2023).

At least 300 copepod individuals were counted and identified per sample using a microscope for detailed examination of specimens when necessary. In addition, the entire samples were examined qualitatively to assess the presence of rare copepod taxa (Table S2). Early developmental stages of copepods were identified to the lowest possible taxonomic level and either grouped with the corresponding adults, if applicable, or reported at a higher taxonomic level (genus or family). Gastropods with coiled shell that could not be reliably identified as either pteropods or gastropod larvae were grouped under Gastropoda. Due to fragmentation of siphonophore colonies, counts of anterior nectophores were used as colony proxies for calycophoran siphonophores. Pneumatophores are commonly used as colony proxies for physonect siphonophores; however, no physonects were encountered in the samples.

Abundance and dry weight values were expressed as individuals per square meter (ind. m^−2^) and grams per square meter (g m^−2^), respectively, in order to ensure fair comparison of mesozooplankton standing stock from deeper (sampling depth: 0–200 m) and shallow stations (sampling depth: 0–30 m). To allow comparison with other studies, we also provide, as supplementary material, density expressed as ind. m^−3^ (Table S1).

### Data processing

All statistical analyses and plotting of the results were carried out using R programming language (R Core Team, 2024) on R studio software (RStudio Team, 2024). Maps and plots were conducted with the package “ggplot2” (Wickham, 2016).

Contour maps of mean temperature, mean salinity and integrated Chl-a at the upper 25 m depth were produced through interpolation using the Multilevel B-Spline Approximation (MBA) method (Lee *et al*., 1997) implemented via the “MBA” package (Finley *et al*., 2024). The differentiation of the two subsystems (W-GCLME & C-GCLME), based on the environmental parameters used for the contour maps, was tested by permutational multivariate analysis of variance (PERMANOVA, Anderson, 2001, 2017), using the Euclidean distances of prior normalized data and 9999 implemented permutations, via the “vegan” package (Oksanen *et al*., 2025). The same approach was applied within the C-GCLME to compare areas located east and west of the Cape Three Points.

A two-way analysis of variance (ANOVA) was conducted to investigate differences in total mesozooplankton abundance and dry weight, considering two factors: subsystem (W-GCLME vs C-GCLME) and sampling layer (30, 100 and 200 m). Prior to analysis, datasets were assessed for normality (Shapiro–Wilk test, α = 0.05) and homogeneity of variance (Levene’s test, α = 0.05); the data were log-transformed where the assumption of normality was not met.

Spatial variability in the copepod and diplostracan assemblage was explored through multivariate analyses, as these were the groups identified to the lowest taxonomic resolution across samples. The abundance data of all taxa listed in Table II (excluding the unidentified Calanoida and Harpacticoida) were square root transformed to reduce the effect of the dominant taxa and enhance the contribution of moderately abundant taxa. Bray–Curtis dissimilarity index was then calculated between every pair of samples, and the resulting dissimilarity matrix was subjected to hierarchical agglomerative clustering coupled with a group average linkage (Clarke *et al.*, 2014) and principal coordinate analysis (PCoA, Legendre and Anderson, 1999). The statistical significance of sample clustering was tested with the similarity profile routine (SIMPROF) in 1% significance level (Clarke *et al*., 2008) applying the *simprof* function of the package “clustsig” (Whitaker and Christman, 2014). To detect the taxa contributing up to 80% in the similarity of the clustered samples, similarity percentage analysis (SIMPER) was performed (Clarke *et al*., 2014).

Finally, diversity indices (taxonomic richness, Shannon-Weiner-H and Pielou’s evenness) were also computed based on the copepod and diplostracan genera identified in the collections (Shannon and Weaver, 1949; Pielou, 1966). Multivariate analyses and calculation of the diversity indices were conducted with the package “vegan” (Oksanen *et al*., 2025). The means of total copepod and diplostracan abundance, as well as diversity indices among the groups of stations identified by Cluster analysis, were compared using one-way ANOVA, followed by Tukey’s post-hoc tests whenever differences were found between any set of data. Similarly, differences in the means of the diversity indices between the two subsystems were also explored.

Copepod and diplostracan community structure was further associated with the environmental parameters using the constrained method of distanced-based redundancy analysis (dbRDA, Legendre and Anderson, 1999). This analysis can be based on any distance or similarity matrix, including the Bray–Curtis dissimilarity matrix, using PCoA (Legendre and Anderson, 1999). Environmental data were normalized and checked for collinearity with variance inflation factors (VIFs), using a preselected threshold of two, which indicates no significant collinearity (Zuur *et al.*, 2010). The variables tested were mean temperature, mean salinity and integrated Chl-a at the upper 25 m depth, as well as sampling depth. To determine the best combination of environmental parameters explaining the variation in zooplankton data, we used the *ordistep*function from the “vegan” package, employing a forward selection procedure and the Akaike information criterion (AIC). The significance of the model was assessed through permutations (*n* = 9999).

## RESULTS

### Hydrography

**Fig. 2:**
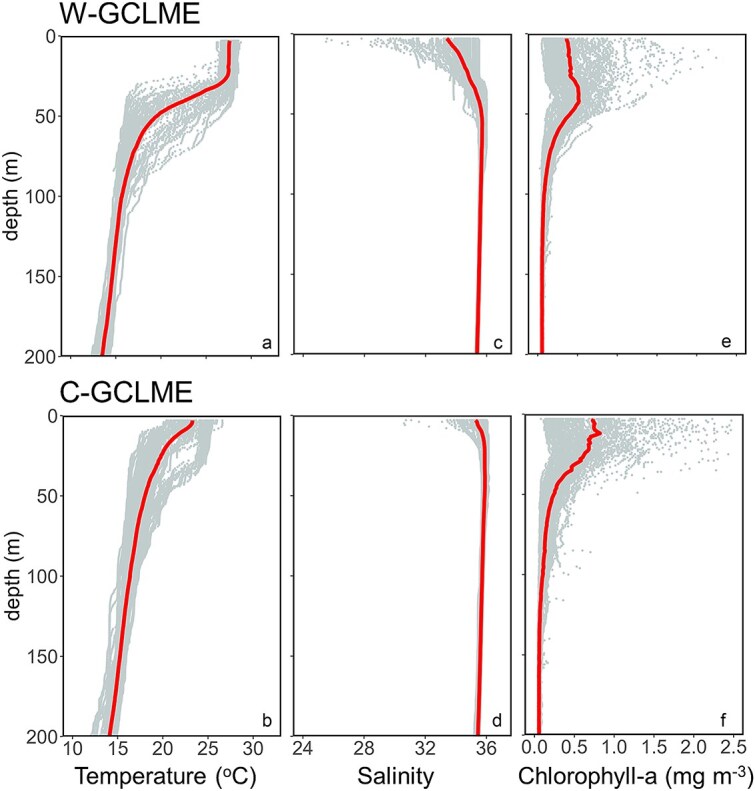
Vertical profiles of temperature (a, b), salinity (c, d) and chlorophyll-*a* (e, f) in the upper 200 m of the water column across W-GCLME (a, c, e) and C-GCLME (b, d, f). Profiles are based on data from 245 CTD stations (W-GCLME: 120, C-GCLME: 125). Mean profile for each subsystem is shown in red.

**Fig. 3:**
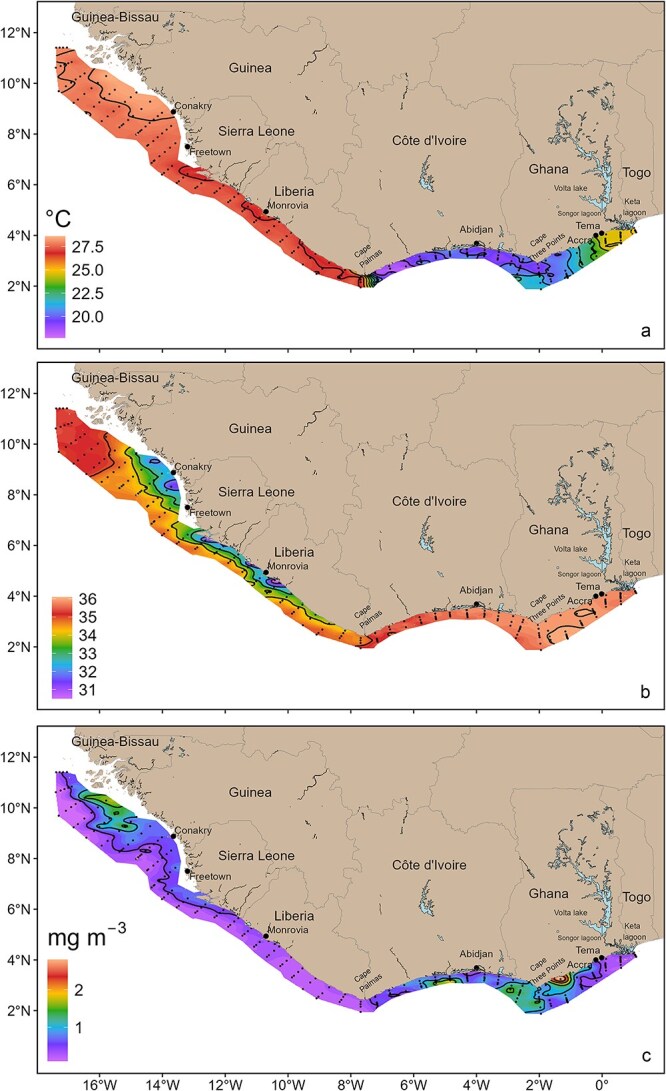
Spatial distribution of (a) mean temperature, (b) mean salinity and (c) integrated chlorophyll-*a* in the upper 25 meters of the water column across GCLME, based on data from 245 CTD stations.

Figure 2 shows the vertical profiles of temperature, salinity and Chl-a concentrations recorded in the upper 200 m of the GCLME subsystems considered in the present study. Water column structure presented strong differences between the two subsystems during the study period. In the W-GCLME, a distinct thermocline developed between ca. 30 and 70 m depth, with a deep Chl-a maximum at 30–50 m depth. In contrast, the water column in the C-GCLME was generally well mixed at most stations, with maximum Chl-a concentrations closer to the surface. Interestingly, a distinct group of stations within C-GCLME exhibited relatively warmer and stratified waters (Fig. 2b); most of these stations located in the easternmost part of the region, as shown in Fig. 3a. Salinity profiles also differed between the two subsystems, with the C-GCLME generally exhibiting slightly higher surface salinity and a less pronounced vertical salinity gradient in the upper layers compared to the W-GCLME.

The horizontal spatial distribution of hydrographic variables within the upper 25 m was also significantly different when the two subsystems (W-GCLME and C-GCLME) were compared (Fig. 3; PERMANOVA, Pseudo-F = 226.42, *P* < 0.001). Overall, the C-GCLME was characterized by lower temperatures (mean ± SE: 21.33 ± 0.19°C), higher salinity (35.71 ± 0.02) and cores of high Chl-a values (0.71 ± 0.04 mg m^−3^) within the upper 25 m water column. In contrast, the W-GCLME exhibited generally warmer (27.5 ± 0.04°C), less saline (34.16 ± 0.1) and less productive waters (Chl-a: 0.4 ± 0.03 mg m^−3^); only stations in the northern coastal area of the W-GCLME were characterized by low salinity and high Chl-a waters.

Moreover, hydrographic conditions within the C-GCLME differed significantly between areas located west and east of Cape Three Points (Fig. 3; PERMANOVA, pseudo-F = 42.94, *P* < 0.001). While salinity differences were relatively small (35.59 ± 0.04 west vs 35.86 ± 0.02 east), the western area was characterized by lower temperatures (19.87 ± 0.13°C west vs 23.02 ± 0.23°C east) and higher Chl-a concentrations (0.85 ± 0.05 mg m^−3^ west vs 0.54 ± 0.05 mg m^−3^ east) compared to the eastern area.

### Mesozooplankton abundance and biomass

**Fig. 4:**
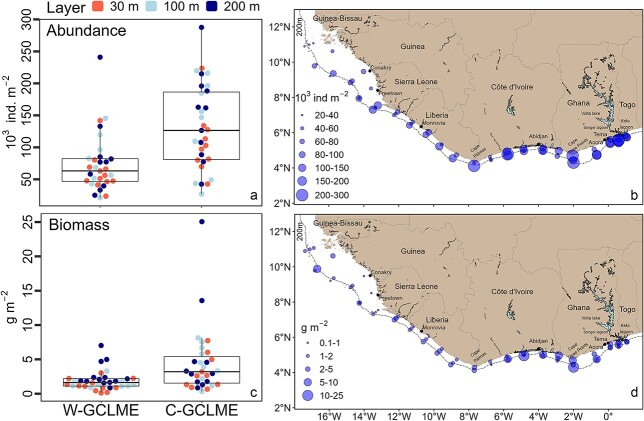
Boxplots and spatial distributions of the total mesozooplankton abundance (individuals m^−2^) (a, b) and biomass, expressed as dry weight (g m^−2^) (c, d), for the W-GCLME (31 stations for abundance, 34 stations for dry weight) and C-GCLME (31 stations for both abundance and dry weight). Data are shown for the different sampling layers: 30, 100 and 200 m.

Mesozooplankton abundance ranged from 21 173 ind. m^−2^ to 287 520 ind. m^−2^ across the entire study area, peaking in the C-GCLME subsystem, particularly at stations deeper than 100 m (Fig. 4a). The dry weight of the samples followed a horizontal pattern similar to that of abundance, ranging from 0.1 to 25.1 g m^−2^ (Fig. 4b). The results showed that mean mesozooplankton abundance in the C-GCLME subsystem was significantly higher (ca. 1.8 times) compared to the W-GCLME (F = 15.97, *P* < 0.001; Fig. 4c, Table S1); the biomass was also significantly higher (ca. 2.3 times) within the C-GCLME (F = 11.56, *P* < 0.01; Fig. 4d, Table S1). Differences among sampling layers were found significant only in the case of biomass (F = 3.83, *P* < 0.05). No significant interaction was found between subsystem and layer for either abundance (F = 0.09, *P* = 0.91), or biomass (F = 0.77, *P* = 0.47).

### Mesozooplankton assemblage—associations with the environment

In total, 16 holoplanktonic and 7 meroplanktonic major groups were identified, with copepods being the most abundant group (Table I). On average, copepods constituted 77.9 ± 1.9% and 81.1 ± 1.6% (mean ± SE) of the total mesozooplankton abundance in the W-GCLME and C-GCLME, respectively. Other holoplanktonic groups such as appendicularians, chaetognaths, ostracods and diplostracans, followed in rank order comprising only a small part of the community (Table I). All major groups were more abundant in the C-GCLME, although the difference was statistically significant only for copepods (F = 18.17, *P* < 0.001). An exception was ostracods, which were slightly more abundant in W-GCLME (Fig. S2).

**Table I:** Mean abundance (ind. m^-2^) and mean relative density (%) of mesozooplankton groups identified in the W-GCLME (*N* = 31) and the C-GCLME (*N* = 31). The standard error is provided in parentheses; (*N*: number of stations)

Taxon	Mean abundance		Relative density (%)	
	W-GCLME	C-GCLME	W-GCLME	C-GCLME
**Holoplankton**				
Copepoda	58 285.23 (7260.6)	105 178.58 (9339.61)	77.9	81.1
Calanoida	35 967.22 (4461.09)	59 668.39 (5208.95)	48.06	46.75
Cyclopoida	19 835.19 (2602.31)	38 058.58 (3876.1)	26.76	29.21
Harpacticoida	2482.83 (525.51)	7451.61 (1552.01)	3.06	5.13
				
Amphipoda	119.64 (24.51)	185.81 (59.63)	0.2	0.1
Appendicularia	4907.44 (623.35)	10 190.19 (1709.27)	7.3	7.5
* Firtillaria* sp.	254.4 (77.7)	104.52 (53.01)	0.40	0.07
* Oikopleura* sp.	4653.04 (603.52)	10 085.68 (1707.24)	6.91	7.42
Chaetognatha	2105.25 (285.51)	3371.61 (562.98)	3	2.4
Diplostraca	196.13 (165.16)	3401.81 (787.84)	0.3	2.3
* Peniliaavirostris*	172.9 (149.88)	3066.32 (746.76)	0.27	2.03
* Pseudoevadne tergestina*	23.23 (17.08)	335.48 (99.3)	0.03	0.25
Doliolida	458.32 (104.95)	1725.68 (691.06)	0.7	1.1
Euphausiacea	586.58 (167.54)	558.71 (148.01)	0.8	0.4
Gastropoda^1^	974.71 (325.25)	1005.42 (248.88)	1.2	0.8
Isopoda	29.79 (13.63)	6.97 (4.88)	0	0
Lucifer	196.12 (58.04)	206.44 (127.52)	0.4	0.1
Medusozoa	3.1 (3.1)	123.87 (50.85)	0	0.1
Mysida	20.65 (16.16)	0 (0)	0	0
Ostracoda	2735.78 (883.21)	1970.58 (434.99)	4.5	1.5
Pteropoda^2^	140.9 (77.2)	248.26 (66.98)	0.2	0.2
Salpida	23.23 (17.08)	0 (0)	0	0
Siphonophorae	100.1 (27.28)	398.45 (90.98)	0.1	0.3
**Meroplankton**				
Bivalvia	627.33 (235.23)	476.39 (150.26)	1	0.3
Cirripedia	206.71 (83.32)	1224.52 (466.63)	0.4	0.8
Decapoda	385.72 (69. 96)	326.2 (89.52)	0.6	0.2
Fish egg	54.45 (35.2)	101.94 (63.5)	0.1	0.1
Fish larvae	101.04 (24.28)	85.16 (43.89)	0.2	0
Polychaeta	1048.78 (751.49)	701.68 (182.15)	0.9	0.5
Stomatopoda	1.94 (1.94)	0 (0)	<0.01	0

^1^ includes coiled shell individuals that can be either holoplanktonic gastropods or gastropod larvae

^2^ includes cone-shaped Thecosomata.

A total of 104 copepod taxa were identified in the routine subsample counts (W-GCLME: 85; C-GCLME: 92; Table II). Among these, 73 taxa were common to both subsystems, while 12 taxa were recorded exclusively in the W-GCLME and 19 taxa exclusively in the C-GCLME. Additionally, 26 rare or taxonomically challenging taxa were detected during the exhaustive scanning of the entire sample (Table S2). Calanoids, represented by 78 taxa, were the dominant copepod order, followed by cyclopoids (20 taxa) and harpacticoids (6 taxa) (Table II). All copepod orders and most of their taxa occurred in higher abundances in the C-GCLME (Table I, Table II). However, the diversity indices based on copepod and diplostracan genera did not differ statistically between the two subsystems (Table S3).

**Table II:** Mean abundance (ind. m^-2^) and mean relative contribution (%) of copepod and diplostracan taxa identified in the samples collected from the W-GCLME (*N* = 31) and the C-GCLME (*N* = 31). The standard error is provided in parentheses; (*N*: number of stations)

Taxon	Mean abundance		Mean relative contribution (%)	
	W-GCLME	C-GCLME	W-GCLME	C-GCLME
**Calanoida**				
*Acartia danae*	85.68 (30.35)	340.65 (91.93)	0.2	0.3
*Acartia negligens*	16.52 (11.86)	7.74 (7.74)	<0.1	<0.1
*Acartia* spp.	37.71 (12.42)	198.71 (71.06)	0.1	0.2
*Acrocalanus* spp.	733.94 (222.9)	111.48 (42.72)	0.9	0.1
*Aetideopsis* sp.	−	7.74 (7.74)	−	<0.1
*Aetideus* spp.	41.81 (19.97)	112.26 (39.72)	0.1	0.1
*Calanoides natalis*	12.9 (9.16)	4368 (1189.85)	<0.1	4.1
*Calocalanus pavo*	850.63 (194.8)	49.03 (23.07)	1.4	<0.1
*Calocalanus plumulosus*	3.93 (2.76)	−	<0.1	−
*Calocalanus* spp.	828.86 (216.42)	1645.94 (346.49)	1.3	1.6
*Candacia curta*	7.74 (7.74)	15.48 (15.48)	<0.1	<0.1
*Candacia pachydactyla*	15.48 (10.77)	−	<0.1	−
*Candacia* spp.	54.19 (27.61)	127.74 (32.89)	0.1	0.1
*Centropages bradyi*	−	15.48 (15.48)	−	<0.1
*Centropages chierchiae*	2.82 (2.81)	4763.1 (1021.63)	<0.1	4.4
*Centropages velificatus*	1046.33 (198)	955.87 (219.19)	2.6	0.8
*Centropages violaceus*	18.58 (11.06)	−	<0.1	−
*Clausocalanus furcatus*	3316.22 (967.99)	481.03 (134.14)	4.7	0.5
*Clausocalanus jobei*	101.81 (34.08)	849.29 (226.14)	0.2	0.7
*Clausocalanus mastigophorus*	25.44 (17.12)	85.16 (39.6)	<0.1	0.1
*Clausocalanus parapergens*	17.55 (13.26)	206.45 (78.5)	<0.1	0.2
*Clausocalanus pergens*	62.45 (30.09)	42.58 (31.75)	0.1	<0.1
*Clausocalanus* spp.	5372.56 (1341.66)	4637.16 (865.3)	7.2	4
*Ctenocalanus vanus*	251.75 (80.23)	1872 (406.94)	0.6	1.5
*Delibus nudus*	783.16 (363.65)	60.65 (27.32)	0.9	0.1
*Diaixis pygmaea*	28.39 (13.83)	154.06 (69.25)	0.1	0.3
*Euaugaptilus hecticus*	−	15.48 (15.48)	−	<0.1
*Euaugaptilus* sp.	−	15.48 (15.48)	−	<0.1
*Euchaeta paraconcinna*	2044.67 (365.13)	915.1 (153.25)	3.8	1
*Euchaeta-Paraeuchaeta* spp.	−	500.65 (355.12)	−	0.3
*Haloptilus longicornis*	10.84 (6.36)	69.68 (27.7)	<0.1	<0.1
*Haloptilus* spp.	−	58.06 (26.61)	−	<0.1
*Heterorhabdus guineanensis*	16.52 (11.86)	6.19 (6.19)	<0.1	<0.1
*Heterorhabdus* sp.	6.88 (5.39)	19.35 (11.26)	<0.1	<0.1
*Labidocera nerii*	16.89 (16.89)	−	0.1	−
*Labidocera scotti*	3.1 (3.1)	−	<0.1	−
*Labidocera* spp.	72.41 (20.76)	−	0.1	−
*Lucicutia clausi*	−	21.68 (16.48)	−	<0.1
*Lucicutia flavicornis*	116.73 (44.35)	139.35 (50.73)	0.2	0.1
*Lucicutia* spp.	115.61 (44.11)	99.87 (37.78)	0.2	0.1
*Mecynocera clausi*	21.34 (9.71)	173.42 (63.92)	0.1	0.2
*Microcalanus pygmaeus*	160 (100.1)	89.03 (63.44)	0.2	0.1
*Nannocalanus minor*	214.19 (72.2)	175.48 (59.87)	0.3	0.1
*Paivella inaciae*	−	34.84 (21.69)	−	<0.1
*Paracalanus aculeatus* complex	301 (79.32)	310.45 (76.55)	0.5	0.3
*Paracalanus parvus* complex	2655.55 (235.61)	4740.65 (712.08)	5.7	4.7
*Paracalanus* spp.	7343.08 (1038.28)	13 005.68 (1530.95)	13.4	13.4
*Paraeuchaeta hebes*	−	69.68 (43.38)	−	<0.1
*Pareucalanus sewelli*	46.45 (39.22)	5.16 (5.16)	<0.1	<0.1
*Parvocalanus scotti*	1960.26 (1266.3)	1950.45 (693.97)	3.4	2
*Pleuromamma abdominalis*	10.84 (8.24)	−	<0.1	−
*Pleuromamma gracilis*	28.9 (15.16)	77.42 (30.25)	0.1	0.1
*Pleuromamma* spp.	77.94 (33.33)	260.65 (82.87)	0.1	0.3
*Pontellina* sp.	12.9 (9.16)	−	<0.1	−
*Pontellopsis brevis*	−	15.48 (15.48)	−	<0.1
*Pseudodiaptomus serricaudatus*	263.84 (146.01)	44.39 (22.24)	0.6	0.1
*Rhincalanus cornutus*	7.74 (7.74)	279.23 (85.44)	<0.1	0.2
*Scaphocalanus curtus*	−	40.26 (19.16)	−	0.1
*Scaphocalanus* spp.	−	230.71 (75.89)	−	0.3
*Scolecithricella abysallis*	5.16 (5.16)	−	<0.1	−
*Scolecithricella profunda*	−	46.45 (46.45)	−	<0.1
*Scolecithricella* spp.	11.35 (6.56)	−	<0.1	−
*Scolecithrix bradyi*	18.58 (11.06)	15.48 (15.48)	<0.1	<0.1
*Scolecithrix danae*	33.2 (17.7)	23.23 (17.08)	<0.1	<0.1
*Scolecithrix* spp.	63.41 (33.09)	28.39 (17.62)	0.1	<0.1
*Scolecitrichopsis ctenopus*	20.65 (11.71)	54.19 (30.9)	<0.1	<0.1
*Scolecitrichopsis* spp.	−	15.48 (15.48)	−	<0.1
*Scolecitrichopsis tenuipes*	37.16 (13.13)	7.74 (7.74)	0.1	<0.1
*Subeucalanus crassus*	7.74 (7.74)	−	<0.1	−
*Subeucalanus monachus*	129.03 (57.79)	132.65 (53.99)	0.1	0.1
*Subeucalanus pileatus*	243.25 (44.6)	174.45 (54.5)	0.5	0.2
*Subeucalanus* spp.	1858 (403.51)	6600.26 (1272.32)	3.4	6.4
*Subeucalanus subtenuis*	9.46 (7.87)	23.23 (17.08)	<0.1	<0.1
*Temora* spp.	1705.29 (547.59)	5054.97 (1050.72)	3.1	5.4
*Temora stylifera*	627.61 (118.27)	738.58 (153.77)	1.6	0.6
*Temora turbinata*	395.35 (169.94)	1199.48 (358.36)	0.7	1.1
*Temoropia mayumbaensis*	163.1 (71.87)	123.87 (44.34)	0.2	0.1
*Undinula vulgaris*	864.67 (182)	206.45 (83.29)	1.5	0.2
Unidentified Calanoida	569.57 (105.18)	755.1 (169.49)	1	0.7
**Cyclopoida**				
*Agetus flaccus*	−	7.74 (7.74)	−	<0.1
*Agetus limbatus*	15.48 (10.77)	7.74 (7.74)	<0.1	<0.1
Corycaeidae	1925.37 (307.81)	3806.71 (492.01)	3.7	3.9
*Corycaeus speciosus*	408.5 (100.09)	131.61 (79.44)	0.6	0.1
*Ditrichocorycaeus africanus*	945.36 (527.86)	1939.1 (564.06)	1.7	1.5
*Farranula gracilis*	1466.57 (413.44)	526.97 (162.67)	2.1	0.5
*Farranula rostrata*	−	7.74 (7.74)	−	<0.1
*Lubbockia squillimana*	31.48 (17.72)	282.32 (73.48)	<0.1	0.3
*Oithona nana*	2078.68 (374.58)	1900.39 (325.83)	4.4	2.2
*Oithona plumifera*	1549.93 (275.53)	2642.58 (585.52)	2.6	2.3
*Oithona* spp.	2715.94 (555.08)	8925.42 (1163.1)	4.3	8.5
*Oncaea-Triconia* spp.	7766.31 (1395.32)	15 467.61 (1799.69)	12.6	14.4
*Onychocorycaeus giesbrechti*	688.19 (109.92)	2008.77 (371.58)	1.4	1.9
*Onychocorycaeus ovalis*	124.17 (42.88)	23.23 (12.95)	0.2	<0.1
*Sapphirina metallina*	−	7.74 (7.74)	−	<0.1
*Sapphirina nigromaculata*	5.16 (5.16)	162.58 (59.3)	<0.1	0.1
*Sapphirina ovatolanceolata*	5.16 (5.16)	−	<0.1	−
*Sapphirina* spp.	52.13 (24.23)	63.23 (24.31)	0.1	0.1
*Urocorycaeus furcifer*	56.77 (25.76)	131.61 (48.31)	0.1	0.1
*Vettoria* spp.	−	15.48 (10.77)	−	<0.1
**Harpacticoida**				
*Clytemnestra-Goniopsyllus* sp.	51.85 (17.24)	186.84 (52.64)	0.1	0.2
*Distioculus minor*	−	38.71 (19.59)	−	<0.1
*Euterpina acutifrons*	1268.13 (494.51)	6789.94 (1497.26)	1.8	5.9
*Macrosetella gracilis*	1070.92 (204.88)	223.23 (74.79)	2	0.2
*Microsetella rosea*	34.14 (17.03)	92.9 (63.62)	0.1	0.1
*Miracia efferata*	30.8 (12.21)	104.52 (55.86)	0.1	0.1
Unidentified Harpacticoida	26.99 (11.25)	15.48 (15.48)	0.1	<0.1
**Diplostraca**				
*Penilia avirostris*	172.9 (149.88)	3066.32 (746.76)	0.27	2.03
*Pseudoevadne tergestina*	23.23 (17.08)	335.48 (99.3)	0.03	0.25

The main copepods with ubiquitous presence in both subsystems were *Centropages velificatus*, *Oithona nana*, *Oithona plumifera*, *Onychocorycaeus giesbrechti*, *Temora stylifera*, *Oncaea-Triconia* spp. and *Paracalanus parvus* species complex (Fig. S3). Copepodites of the genera *Clausocalanus*, *Oithona*, *Paracalanus*, *Subeucalanus* and *Temora* were also ubiquitous and very abundant in both subsystems (Fig. S4). However, other taxa identified in the collections presented distinct spatial distribution patterns. Taxa such as *Calanoides natalis* and *Centropages chierchiae* were present only in the cold upwelling waters of C-GCLME, while others such as *Clausocalanus furcatus*, *Farranula gracilis*, *Macrosetella gracilis* and *Undinula vulgaris*, prevailed in the warmer conditions of W-GCLME (Fig. S5). Some taxa, i.e. *Ditrichocorycaeus africanus*, *Euterpina acutifrons*, *Penilia avirostris* and *Temora turbinata* were present in high abundances in the upwelling waters of C-GCLME, as well as in some stations characterized by low salinity and high Chl-a concentration in W-GCLME (Fig. S6). Other taxa appeared only near estuaries, with the most characteristic example being the calanoids *Parvocalanus scotti* and *Pseudodiaptomus serricaudatus* (Fig. S6).

**Fig. 5:**
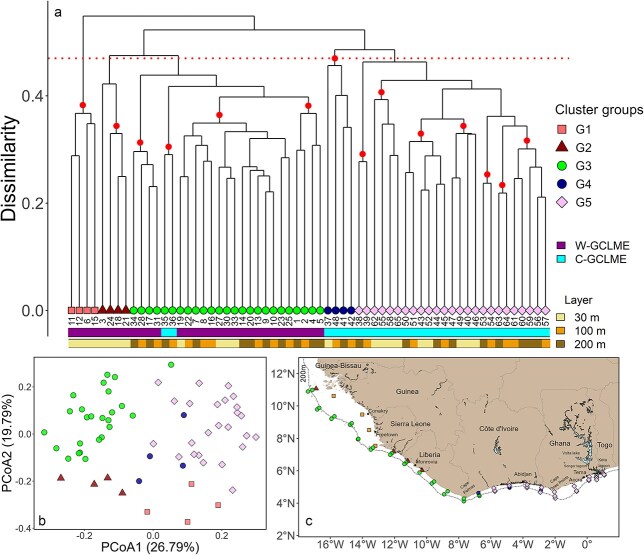
**(**a) Station groups defined by hierarchical cluster analysis of the copepod and diplostracan community. Station numbers, area designation (W-GCLME and C-GCLME), and sampling layer are shown below the dendrogram. Results of the SIMPROF analysis are indicated by red dots atop the cluster branches, (b) PCoA ordination plot of the copepod and diplostracan community, with different colors representing samples assigned to different cluster groups, (c) map of the GCLME with superimposed cluster groups.

**Fig. 6:**
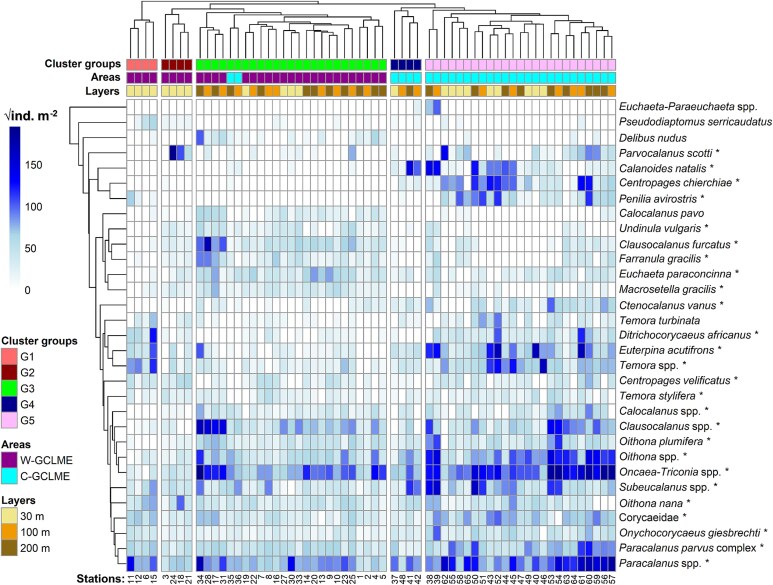
Heatmap of the square root transformed abundances (ind. m^−2^) for taxa contributing >5% to the total copepod and diplostracan abundance in at least one sample. Colored bars indicate station clusters (as shown in Fig. 5a), sampling layers, and the two subsystems: W-GCLME and C-GCLME. Copepod and diplostracan taxa are grouped (using group-average linkage) based on the Bray–Curtis distance matrix of their standardized abundances. Taxa marked with an asterisk (*) contribute to the 80% cumulative similarity identified in the SIMPER analysis (see Table S4).

Cluster analysis based on the abundances of copepod and diplostracan taxa listed in Table II, identified five major groups of sampling stations (Groups: G1–G5) at 47% dissimilarity level (Fig. 5a). Most of these station Groups were clearly separated in the PCoA ordination plot, where the first two axes explained 26.79% and 19.79% of the variability in the original dissimilarity matrix, respectively (Fig. 5b). Groups G1 and G2 occurred only in the coastal waters (depth ca. 30 m) of the W-GCLME; Groups G4 and G5 comprised stations located only in C-GCLME, covering coastal to deeper waters (ca. sampling depths 30–200 m) (Fig. 5a). The rest of the stations, mostly sampled in W-GCLME, formed group G3. Simper analysis revealed notable differences in the copepod and diplostracan assemblage among the five cluster groups (Fig. 6, Table S4) that were also reflected in the diversity indices (Fig. S7). Group G1 stations, located on the Guinea and Sierra Leone plateau (Fig. 5c), stood out distinctly with low taxonomic richness and high evenness (Fig. S7), characterized by dominance of few small sized copepod taxa i.e. *Temora* spp., Corycaeidae, *D. africanus*, *E. acutifrons* and *Paracalanus* spp. (Fig. 6). G2 group was mostly located in the narrow shelf of Liberia and showed particularly high densities of the copepods *P. scotti* and *C. velificatus*, while G3 group (W-GCLME stations plus two sites in the western boundary of the C-GCLME) was characterized by taxa such as *Clausocalanus* spp., *C. furcatus*, *Euchaeta paraconcinna*, *F. gracilis*, *M. gracilis* and *U. vulgaris*. Within C-GCLME, G5 group showed significantly higher total assemblage abundance compared to all other station groups (F = 11.62, *P* < 0.001, Tukey post hoc, *P* < 0.01; Fig. S7) and high densities of *C. natalis*, *C. chierchiae*, *E. acutifrons*, *P. avirostris* as well as absence or lower densities of characteristic taxa recorded in G3 group. G4 group was distinguished from G5 due to higher *C. natalis* densities and the lower densities of most of the other taxa. Interestingly, within the C-GCLME, further spatial differentiation was evident, although at a lower level of dissimilarity. A distinction emerged between C-GCLME stations sampled east and western of Cape Three Points, which was also confirmed by PERMANOVA analysis (Pseudo-F = 5.2975, *P* < 0.001). This was mainly due to a notable presence of *C. natalis* and *Subeucalanus* copepodites in the western part of Cape Three Points (Figs 6, S4 and S5).

**Fig. 7:**
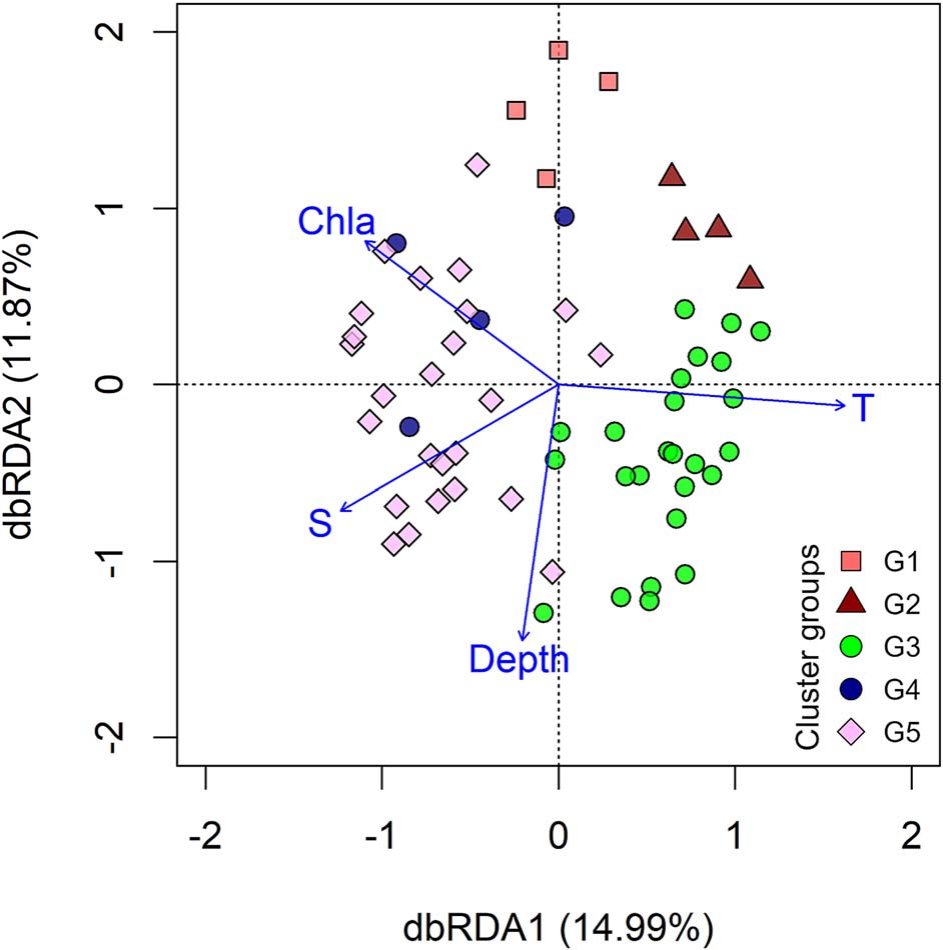
dbRDA biplot (type 2 scaling) of the copepod and diplostracan community. Station groups correspond to the cluster groups (as shown in Fig. 5a). Vectors represent hydrographic parameters including mean temperature (T), mean salinity (S) and integrated chlorophyll-*a* (Chl-a) in the upper 25 m of the water column, and sampling depth (Depth).

The dbRDA indicated that the environmental predictors significantly explained variation in the copepod and diplostracan assemblage structure, accounting for a total of 30.94% of the variability (Table S5). Temperature explained the largest portion of community variability (13.76%), followed by sampling depth (9.40%), Chl-a (4.11%) and salinity (3.67%). The overall dbRDA model and the first two dbRDA axes were statistically significant (overall model: F = 6.38, *P* ≤ 0.001; dbRDA1: F = 12.36, *P* ≤ 0.001; dbRDA2: F = 9.79, *P* ≤ 0.001), with dbRDA1 and dbRDA2 explaining 14.99% and 11.87% of the total variation, respectively (Fig. 7). The ordination revealed that cluster group G3 was associated with higher temperatures and lower Chl-a values, while groups G4 and G5 were related to lower temperatures and higher Chl-a concentrations. Groups G1 and G2 were associated with lower salinity and shallower sampling depths. The selection of variables included in the final model is detailed in Table S6.

## DISCUSSION

The present study, spanning from Guinea-Bissau to Ghana, provides a detailed description of the mesozooplankton distribution across the GCLME. Sampling took place between July and September, coinciding with the West African monsoon and the northward shift of the ITCZ (Vázquez *et al.*, 2023). This period marks the onset of the rainy season in the W-GCLME and the dry season in the C-GCLME (Efon *et al*., 2023), which also corresponds to the major coastal upwelling (Binet *et al*., 1991; Mensah, 1991). The present findings agree with previous reports indicating that the GCLME is a highly productive region, characterized by marked hydrographic contrasts between its subsystems (Binet and Marchal, 1993; Hardman-Mountford and McGlade, 2002a). We observed that C-GCLME and W-GCLME exhibited significant differences in hydrographic parameters i.e. warmer and thermally stratified waters in the W-GCLME and cooler upwelled waters in parts of the C-GCLME (particularly within the western part of C-GCLME region), with a prominent reflection on mesozooplankton stock and copepod/diplostracan assemblage structure.

### Zooplankton distribution patterns within GCLME: central vs western subsystems

Our results showed that the upwelling-influenced areas within C-GCLME, i.e. the coasts of Côte d’Ivoire and Ghana, were characterized by cooler waters, high Chl-a concentration and significantly elevated mesozooplankton stock. These areas exhibited a distinct copepod-diplostracan assemblage, strongly associated with the lower sea surface temperature. The Chl-a concentration observed in the present study is consistent with the high primary productivity documented in the past through field studies (Dandonneau, 1973; Binet, 1983a; Anang, 2000) and satellite ocean color observations (Nieto and Mélin, 2017) of the C-GCLME. Previous studies suggest that the main rainy season (May–June) delivers a substantial pulse of land-derived nutrients to coastal waters (Binet, 1982; Mensah, 1991; Djakouré *et al.*, 2024b), which together with the upwelling-driven nutrient injection in July, stimulates intense plankton bloom within the study area (Mensah, 1991). Therefore, it is likely that the high concentration of Chl-a within C-GCLME was driven by nutrient enrichment from upwelling processes and riverine input associated with the early monsoon rainfall (Binet, 1982; Mensah, 1991; Djakouré *et al.*, 2024b).

The major upwelling period in the C-GCLME is associated with the peak of zooplankton stock (Binet, 1983b; Wiafe *et al*., 2008), dominated by copepods (Binet, 1976, 1977b) and reduced taxonomic diversity (Binet, 1978, 1983b). In this study, copepods were also the main component of the mesozooplankton community (>75% contribution) across the whole GCLME. The contribution of other functional groups, including gelatinous plankton, was generally limited in our collections, with the notable exception of appendicularians. Together with chaetognaths, these groups represented the main non-copepod contributors in both subsystems (ca. 10%), with relative abundances nearly twice those reported for the CCLME during surveys conducted in the same year (Goliat *et al*., 2025). Previous studies in the GCLME area have also identified copepods as the dominant group, followed by appendicularians, chaetognaths and ostracods (Binet, 1977b; Wiafe *et al.*, 2016). In our study, ostracod abundances were similar between subsystems, but their contribution was threefold higher in the W-GCLME (up to 35%) than in the C-GCLME, and sevenfold higher than that reported for the CCLME by Goliat *et al.* (2025). Ostracods are known to benefit from multiple enrichment sources, regardless temperature conditions (Binet, 1977b), and are important component of mesozooplankton in the Guinean and Guinea-Bissau areas (Ndour *et al*., 2018), consistent with our findings. Therefore, our results are in agreement with the general understanding on mesozooplankton dynamics in the GCLME area (Binet, 1977b, 1983b; Thiriot, 1978; Yaqub, 2000; Wiafe *et al.*, 2016), the neighboring canary current large marine ecosystem (CCLME) (Berraho *et al.*, 2015; Ndour *et al.*, 2018; Goliat *et al*., 2025) and tropical Atlantic stratified waters (Binet, 1983b; Champalbert *et al*., 2005; Aka *et al.*, 2018).

Beyond ubiquitous taxa, the copepod/diplostracan assemblage included species strongly associated with upwelling conditions, notably the calanoid copepod *C. natalis.* While earlier studies of the C-GCLME in Ivorian–Ghanaian waters (Bainbridge, 1960; Mensah, 1974; Binet and Suisse de Sainte Claire, 1975), as well as across the wider African Atlantic (Thiriot, 1978) have traditionally reported *Calanoides carinatus*, detailed microscopic examination of our specimens (photos provided in Figs S8 and S9) identified them as *C. natalis*, consistent with more recent morphological and genetic evidence supporting the reclassification of *C. carinatus* to *C. natalis* (Bradford-Grieve *et al.*, 2017; Höring *et al.*, 2017)*.* This lipid-rich species (Bainbridge, 1960; personal observations of orange globules from our samples) is a key energy source for *Sardinella aurita* (Bainbridge, 1960; Binet, 1982) and has been reported to dominate the coastal waters off Côte d’Ivoire and Ghana during major upwelling events, with stage V copepodites ascending from depths > 500 m (Bainbridge, 1960; Binet and Suisse de Sainte Claire, 1975; Teuber *et al*., 2019). It reproduces in diatom-rich surface waters over several generations before retreating to deeper layers, as conditions become unfavorable (Binet and Suisse de Sainte Claire, 1975; Houghton and Mensah, 1978). Unlike *C. natalis*, whose ontogenetic migration is tied to upwelling zones, *C. chierchiae* appeared restricted to the C-GCLME, likely due to its temperature preference. This temperature-sensitive, omnivorous calanoid is thriving between 13 and 20°C (Cruz *et al*., 2013), while it is absent >29°C (Dessier, 1979), and has been documented in several upwelling systems (Dessier, 1979; Binet, 1983b; Sobrinho-Gonçalves *et al*., 2013; Greer *et al*., 2014; Berraho *et al*., 2015; Goliat *et al*., 2025). Its recent northward expansion, likely linked to ocean warming, underscores its potential as an indicator species for assessing climate change impacts (Lindley and Daykin, 2005; Cruz *et al*., 2013).

The seasonal upwelling along the coasts of Côte d’Ivoire and Ghana exhibits spatial variation in both timing and intensity (Hardman-Mountford and McGlade, 2002b). Consisting of two distinct cells, each situated to the east of prominent capes (i.e. Cape Palmas and Cape Three Points), the upwelling initiates east of Cape Three Points and propagates westward, counter to the prevailing eastward flow of the surface Guinea Current (Picaut, 1983; Hardman-Mountford and McGlade, 2002b). In our study, sampling within the C-GCLME began from the western part of the system (Cape Palmas). As a result, stations sampled east of Cape Three Points in early September were likely past the peak of the upwelling season, whereas those sampled east of Cape Palmas in late August coincided more closely with the period of maximum upwelling. This difference was probably reflected in the surface temperature patterns, with lower temperatures recorded between Cape Palmas and Cape Three Points, suggesting seasonal variability and a temporal sampling lag. These observations are consistent with previous studies reporting that the coldest upwelled waters typically occur in August (Wiafe *et al*., 2016). Copepod abundance off Côte d’Ivoire correlates with upwelling, with a two-week lag (Binet, 1976) and previous studies have also described a clear ecological succession during the major upwelling (Binet *et al*., 1972; Binet, 1983b). During the major upwelling, deep-dwelling species are first brought to the surface, followed by large grazers (i.e. *C. natalis*, *Subeucalanus monachus*) dominating once conditions stabilize. As the cold season ends, these species are replaced by smaller omnivorous calanoids and diplostracans feeding on finer particles. This type of succession was likely evidenced in our study i.e. stations sampled east of Cape Three Points with a 22-day lag were clustered separately from east of Cape Palmas, showing lower densities or absence of *C. natalis*, while smaller omnivores (e.g. *C. chierchiae*, *T. turbinata*, *P. avirostris*) peaked in abundance.

The W-GCLME was characterized by strong thermal stratification with a pronounced deep chlorophyll maximum, typical of tropical waters. The lowest abundances of zooplankton in our survey were observed further north, near the Bissagos Islands (~11°N, Guinea-Bissau), presumably reflecting the seasonal relaxation of the Mauritanian Senegalese (12–19°N) upwelling during summer (Cropper *et al*., 2014; Vázquez *et al*., 2023) that leads to lower Chl-a concentrations (Nieto and Mélin, 2017). On the other hand, increased mesozooplankton stock was observed across the shelf of the W-GCLME, particularly near the Sherbro River estuary in southern Sierra Leone, where low-salinity waters and elevated Chl-a levels reflected enhanced nutrient input from river discharge during the Monsoon season (Efon *et al*., 2023). Despite the absence of upwelling, this region is known for high productivity driven by riverine inputs (Binet, 1983a, 1983b), a pattern confirmed in our study. Under these conditions, a less diverse assemblage of opportunistic and estuarine taxa was developed on the Guinea-Sierra Leone Plateau. Notably, neritic-affinity species (Binet, 1983b), were widespread across sites influenced by either upwelling or terrigenous enrichment.

Although ubiquitous species characterized both subsystems, a thermophilic copepod assemblage (i.e. *C. furcatus*, *F. gracilis*, *M. gracilis* and *U. vulgaris*) dominated the warm, oligotrophic waters of the W-GCLME. These species are adapted to low-productive tropical systems (Dessier, 1979; Binet, 1983b). For instance, *C. furcatus* proliferates in oligotrophic surface layers, feeding on microbial and protozooplankton prey items (Mazzocchi and Paffenhöfer, 1998, 1999; Isari *et al*., 2014; Peralba *et al*., 2017), while the herbivorous *U. vulgaris* may capture fast-moving prey with specialized filtering appendages, well suited to the stable warm waters of the W-GCLME (Dessier, 1979; Binet, 1983b; Bode *et al*., 2018). The notable presence of *M. gracilis* is likely linked to its known association with the blooms of cyanobacterium *Trichodesmium*, which typically occur in warm, stratified waters (Bottger-Schnack and Schnack, 1989); this is supported by our concurrent observation of such a bloom during the survey (Konan, unpublished data), highlighting the influence of phytoplankton dynamics on zooplankton assemblages.

### Regional context and comparisons

The results of this study provide a snapshot of the mesozooplankton distribution within the upper 200 m of the water column in the GCLME during the West African Monsoon and major upwelling period. Although lacking the temporal resolution and dynamic insights provided by long-term monitoring, this work offers critical baseline information on the lower trophic levels that support regional productivity. To the best of our knowledge, this is the first study in several decades to present detailed field data on mesozooplankton stocks, major group composition and copepod/diplostracan assemblage structure across distinct GCLME subsystems, using standardized methodology and consistent taxonomic expertise. Such data have been particularly scarce in recent years. Some of these coastal areas were last surveyed over 60 years ago (Bainbridge, 1960; Binet *et al*., 1972; Thiriot, 1978; Binet, 1983b). More recent publications rely on data collected nearly two decades ago, focusing either on limited spatial coverage of the Ivorian continental shelf during the warm season (Aka *et al*., 2018) or on Continuous Plankton Recorder (CPR) deployments between Côte d’Ivoire and Cameroon (Wiafe *et al*., 2016).

Long-term monitoring efforts initiated in the late 1960s for fisheries management have provided valuable insights into mesozooplankton community structure and its seasonal and interannual variability, particularly in relation to the upwelling dynamics (Binet, 1979, 1983b). However, much of the historical data, such as the Ghanaian monitoring records, remain limited to bulk displacement volume measurements (Mensah, 1995; Wiafe *et al*., 2008), lacking the taxonomic resolution needed to fully capture mesozooplankton assemblage complexity and ecological dynamics. Our analysis produced an extensive list of copepod taxa across both subsystems, contributing a valuable update to regional knowledge of copepod diversity. This includes revisions based on recent phylogenetic studies and current taxonomic understanding, which address long-standing uncertainties (e.g. cryptic species) in zooplankton identification (Peters *et al*., 2025). For instance, taxonomic studies in the area and reviews in west Africa (Binet *et al*., 1972; Binet, 1978, 1983b; Thiriot, 1978) broadly used the name of *C. carinatus* for *C. natalis*, the *Centropages furcatus* has been commonly used in place of *C. velificatus* and *P. parvus* has been reported as dominant, despite the presence of cryptic species within this complex (Kasapidis *et al*., 2018).

Comparisons of biomass estimates with previous studies in the region remain challenging because of differences in sampling gear, grid resolution, depth coverage and other methodological inconsistencies. For instance, in Ghana, mesozooplankton biomass has been reported as displacement volume using an ICITA net (mesh size 330 μm) in step-oblique hauls down to depths not exceeding 29 m (Mensah, 1995; Wiafe *et al*., 2008), while Côte d’Ivoire time series at the 35 m shallow coastal station were later adapted to WP2 tows (mesh size of 200 μm) and alternative metrics, such as dry weight and ash-free dry weight, that offer a more realistic estimation of organic biomass (Le Borgne and Binet, 1979). A recent study of the adjacent CCLME, conducted just before the onset of our surveys and using the same sampling methodology (Goliat *et al*., 2025), reported a southward increase in mean zooplankton biomass across different zones (from 0.72 to 2.44 g C m^−2^) with the highest values to be observed between 12–21°N (their Zone D). Assuming that carbon content represents ~40% of dry mass (Omori and Ikeda, 1984; Escribano *et al*., 2007) our values in the W-GCLME with a mean of 1.04 g C m^−2^, are much lower than the neighboring Senegalese-Mauritanian upwelling system and similar to the less productive northern CCLME. In contrast, the upwelled waters of C-GCLME in our study exhibited a mean of 1.78 g C m^−2^, falling within the range observed in the most productive zones of the CCLME, and comparable with the annual means reported for the northern Benguela upwelling system (17.3–23° S; 0.74–3.48 g C m^−2^) (Huggett *et al*., 2009; Fernández-Urruzola *et al.,* 2014; Martin *et al*., 2015). Differences in light conditions during sampling may have led to some overestimation of biomass at deeper stations (bottom depth > 500 m), due to diel migration of taxa such as euphausiids. However, these taxa were rare in our samples and are not efficiently sampled by the WP2 net as also discussed by (Goliat *et al*., 2025). On the other hand, the copepod and diplostracan assemblages should not have been influenced by light conditions, as no pronounced DVM patterns have been observed for the majority of copepod taxa in our samples (Binet, 1977a).

Plankton net mesh size is known to strongly influence estimates of zooplankton diversity, abundance and biomass across a wider range of marine systems including temperate (e.g. Hopcroft *et al*., 2001), oligotrophic subtropical (e.g. Calbet *et al*., 2001; Zervoudaki *et al*., 2007) and tropical waters (e.g. Hopcroft *et al*., 2001; Garcia *et al*., 2021). The relatively coarse mesh sizes used in previous GCLME studies (200 and 330 μm), as well as in the present study (180 μm), likely under sample early development stages or adults of small-bodied copepod species, leading to underestimation of biomass and potentially biasing the representation of community structure. Within the GCLME, the magnitude of this underestimation probably varies according to environmental and seasonal conditions. For instance, a stronger bias would be expected under stratified warm-water conditions, where small copepod species and juvenile stages tend to become more abundant. In contrast, during productive periods associated with upwelling and elevated phytoplankton biomass, the larger size fraction is expected to dominate the community.

At the same time, the use of finer mesh sizes may be constrained by rapid net clogging and reduced filtration efficiency, potentially affecting quantitative reliability. This reflects an inherent trade-off between capturing smaller size classes and maintaining consistent sampling performance across contrasting environmental conditions. Although the standardized use of a 180 μm mesh provides a robust baseline for assessing the mesozooplankton fraction > 180 μm and enables future temporal comparisons, upcoming monitoring efforts should also incorporate finer-mesh nets targeting smaller size fractions to better resolve small-bodied taxa and improve estimates of total zooplankton biomass and biodiversity.

The GCLME supports highly productive fisheries and several key commercial fish species, including small pelagic (e.g. sardinellas and anchovies), large pelagic (e.g. tuna species), and demersal species (e.g. sciaenid and sparid communities) (Mensah and Quaatey, 2002; Koranteng, 2002a, 2002b)*.* Many of these species depend on mesozooplankton as a primary food source, either during their early life stages or throughout adulthood, and major upwelling period offers an ideal environmental window for their reproduction and feeding. While historical collapses of *S. aurita* fisheries in the C-GCLME were mainly attributed to overfishing (Mensah, 1995), more recent trends indicate that declining mesozooplankton stocks and shifts in community composition, potentially driven by rising sea temperatures and climate change (Wiafe *et al*., 2008), may increasingly compromise the resilience of these fisheries. The combined effects of climate warming, the growing frequency of marine heatwaves (Koné *et al**.,* 2022) and various forms of marine pollution are also impacting the survival and other vital rates of food-web components (Acheampong *et al*., 2021; Hernández Ruiz *et al*., 2021; Albani *et al*., 2023; Adhiambo *et al*., 2025), altering community structure and thereby affecting overall ecosystem functioning.

Zooplankton organisms are closely linked to essential ecosystem services (Grigoratou *et al*., 2025), and their biomass and diversity serve as essential ocean and climate variables that are commonly used to evaluate fisheries potential and ecosystem health (Miloslavich *et al*., 2018). While the challenges and limitations associated with plankton observations are well documented and apply globally (Grigoratou *et al*., 2022), they are particularly acute in Africa due to the absence of systematic monitoring, limited technical capacity and a shortage of taxonomic expertise. Several ecosystems still lack baseline information related to the status of their components and their capacity to sustain anthropogenic impacts. Monitoring lower trophic levels through harmonized methodologies is therefore critical in GCLME, not only to enable meaningful comparisons across regions, but also to understand and predict how ongoing climate change may impact marine productivity. This is especially important in tropical waters, where organisms already live near their upper thermal limits (Nguyen *et al*., 2011) and ecosystems are additionally threatened by intense pollution including plastics, oil spills, chemical discharges and gold mining activities.

## CONCLUSION

This study highlights the strong influence of regional hydrographic forcing on zooplankton distribution and copepod/diplostracan assemblage within the GCLME. Seasonal upwelling in the central subsystem promotes higher mesozooplankton abundance and biomass and supports communities dominated by upwelling-associated and opportunistic copepods. In contrast, the western subsystem, characterized by stratified, low-salinity waters driven by river discharge, supports thermophilic and opportunistic copepod species. These contrasting environmental settings define two ecologically distinct subsystems within the GCLME. The baseline information provided here contributes to a better understanding of plankton-driven food web dynamics and offers a reference for future ecosystem assessments in this highly productive region.

## Supplementary Material

Anadoli_et_al_supplementary_material_revised_fbag050

## Data Availability

The data were collected within the framework of the EAF-Nansen Programme and can be requested through the Food and Agriculture Organization of the United Nations (FAO) webpage (https://www.fao.org/in-action/eaf-nansen/data/data-access/en).

## References

[ref1] Acheampong, E., Mantey, P. and Weremfo, A. (2021) Potential impact of marine heatwaves on selected phytoplankton adapted to the Gulf of Guinea during stable hydrographic periods. Afr. J. Mar. Sci., 43, 77–86. 10.2989/1814232X.2021.1879267.

[ref2] Adhiambo, R., Mensah, P. K., Koomson, A. and Acheampong, E. (2025) Navigating stress: impacts of temperature, polycyclic aromatic hydrocarbons, and heavy metals on the diatom Thalassiosira weissflogii adapted to tropical waters of the Gulf of Guinea. Aquat. Toxicol., 284, 107388. 10.1016/j.aquatox.2025.107388. 40318463

[ref3] Aka, N. M., Etile, R. N., Joany, T. and N’Da, K. (2018) Peuplement zooplanctonique du plateau continental ivoirien: diversité, abondance et biomasse. Int. J. Biol. Chem. Sci., 12, 129–140. 10.4314/ijbcs.v12i1.10.

[ref4] Albani, G., Asiedu, D., Abrokwah, S., Jónasdóttir, S. H., Nielsen, T. G., Acheampong, E., Ruiz, L. H., Ekumah, B. et al. (2023) Synergistic and additive effects of microplastic, nickel and pyrene on survival, reproduction, and egestion of a tropical copepod. Aquat. Toxicol., 265, 106737. 10.1016/j.aquatox.2023.106737. 37939499

[ref5] Anang, E. R. (2000) The seasonal cycle of the phytoplankton in the coastal waters of Ghana. Mar. Pollut. Bull., 62, 33–45. 10.1007/BF00012560.

[ref6] Anderson, M. J. (2001) A new method for non-parametric multivariate analysis of variance. Austral Ecol., 26, 32–46. 10.1111/j.1442-9993.2001.01070.pp.x.

[ref7] Anderson, M. J. (2017) Permutational multivariate analysis of variance (PERMANOVA). In Balakrishnan, N., Colton, T., Everitt, B., Piegorsch, W., Ruggeri, F., and Teugels, J. L. (eds), Wiley StatsRef: Statistics Reference Online, John Wiley & Sons, Ltd., Hoboken, NJ, USA, pp. 1–15. 10.1002/9781118445112.stat07841c

[ref8] Bainbridge, V. (1960) Occurrence of *Calanoides carinatus* (Kroyer) in the plankton of the Gulf of Guinea. Nature, 188, 932–933. 10.1038/188932a0.

[ref9] Bakun, A. (1978) Guinea current upwelling. Nature, 271, 147–150. 10.1038/271147a0.

[ref110] Berraho, A., Somoue, L., Hernández-León, S., and Valdés, L. (2015) Zooplankton in the Canary Current Large Marine Ecosystem. In Valdés, L. and Déniz-González, I. (eds), Oceanographic and Biological Features in the Canary Current Large Marine Ecosystem. IOC-UNESCO, Paris, pp. 183–195. http://hdl.handle.net/1834/9188

[ref10] Binet, D. (1976) Biovolumes et poids secs zooplanctoniques en relation avec le milieu pélagique au-dessus du plateau Ivoirien. Cah. ORSTOM, sér. Océanogr., 14, 301–326.

[ref11] Binet, D. (1977a) Cycles biologiques et migrations ontogéniques chez quelques copépodes pélagiques des eaux ivoiriennes. Cah. ORSTOM, sér. Océanogr., 15, 111–138.

[ref12] Binet, D. (1977b) Grands traits de l’écologie des principaux taxons du zooplancton ivoirien. Cah. ORSTOM, sér. Océanogr., 15, 89–109.

[ref13] Binet, D. (1978) Analyse globale des populations de copépodes pélagiques du plateau continental ivoirien. Cah. ORSTOM, sér. Océanogr., 16, 19–61.

[ref14] Binet, D. (1979) Le zooplancton du plateau continental Ivoirien. Essai de synthèse écologique. Oceanol. Acta, 2, 397–410.

[ref15] Binet, D. (1982) Influence des variations climatiques Sur la pêcherie des Sardinella aurita ivoiro-ghanéennes: relation sécheresse-surpêche. Oceanologica, 5, 443–452.

[ref16] Binet, D. (1983a) Phytoplancton et production primaire des régions côtières à upwellings saisonniers dans le Golfe de Guinée. Océanogr. Trop., 18, 331–355.

[ref17] Binet, D. (1983b) Zooplancton des régions côtières à upwellings saisonniers du Golfe de Guinée. Océanogr. Trop., 18, 357–380.

[ref18] Binet, D. (1995) Hypotheses accounting for the variability of *Sardinella* abundance in the northern Gulf of Guinea. In Cury, P. and Roy, C. (eds.), Dynamics and Uses of Sardinella Resources from Upwelling off Ghana and Côte d’Ivoire, ORSTOM editions, Paris, pp. 98–133.

[ref19] Binet, D., de Sainte, S. and Claire, E. (1975) Le Copépode planctonique *Calanoides carinatus*. Répartition et cycle biologique au large de la Côte d’Ivoire. Cah. ORSTOM, sér. Océanogr., 13, 15–30.

[ref20] Binet, D., Gaborit, M. and Roux, M. (1972) Copépodes pélagiques du plateau ivoirien. Utilisation de l’analyse des correspondances dans l’étude des variations saisonnières. Doc. Scient. Centre Rech. Océanogr. Abidjan, **3**, 47–79 http://hdl.handle.net/1834/24876.

[ref21] Binet, D. and Marchal, E. (1993) The large marine ecosystem of shelf areas in the Gulf of Guinea: long-term variability induced by climatic changes. In Sherman, K., Alexander, L. M. and Gold, B. D. (eds.), Large Marine Ecosystems: Stocks, Mitigation and Sustainability, AAAS Press, Washington, DC, USA, pp. 104–118.

[ref22] Binet, D., Marchal, E. and Pezennec, O. (1991) *Sardinella aurita* de côte-d’ivoire et du Ghana: fluctuations halieutiques et changements climatiques. In Cury, P. and Roy, C. (eds.), Pêcheries Ouest-Africaines. Variabilité, Instabilité et Changement, ORSTOM editions, Paris, pp. 320–342.

[ref23] Bode, M., Hagen, W., Cornils, A., Kaiser, P. and Auel, H. (2018) Copepod distribution and biodiversity patterns from the surface to the deep sea along a latitudinal transect in the eastern Atlantic Ocean (24°N to 21°S). Prog. Oceanogr., 161, 66–77. 10.1016/j.pocean.2018.01.010.

[ref24] Boltovskoy, D., Gibbons, M. J., Hutchìngs, L. and Binet, D. (1999) General biological features of the South Atlantic. In Boltovskoy, D. (ed.), South Atlantic Zooplankton, Backhuvs Publishers, Leiden, pp. 1–42.

[ref25] Bottger-Schnack, R. and Schnack, D. (1989) Vertical distribution and population structure of *Macrosetella gracilis* (Copepoda: Harpacticoida) in the Red Sea in relation to the occurrence of *Oscillatoria* (*Trichodesmium*) spp. (cyanobacteria). Mar. Ecol. Prog. Ser., 52, 17–31. 10.3354/MEPS052017.

[ref26] Boxshall, G. A. and Halsey, S. H. (2004) An Introduction to Copepod Diversity, The Ray Society, London.

[ref27] Bradford-Grieve, J. M., Blanco-Bercial, L. and Prusova, I. (2017) *Calanoides natalis* Brady, 1914 (Copepoda: Calanoida: Calanidae): identity and distribution in relation to coastal oceanography of the eastern Atlantic and western Indian oceans. J. Nat. Hist., 51, 807–836. 10.1080/00222933.2017.1296198.

[ref28] Calbet, A., Garrido, S., Saiz, E., Alcaraz, M. and Duarte, C. M. (2001) Annual zooplankton succession in coastal NW mediterranean waters: the importance of the smaller size fractions. J. Plankton Res., 23, 319–331. 10.1093/plankt/23.3.319.

[ref29] Champalbert, G., Pagano, M., Kouamé, B. and Riandey, V. (2005) Zooplankton spatial and temporal distribution in a tropical oceanic area off West Africa. Hydrobiologia, 548, 251–265. 10.1007/s10750-005-5194-y.

[ref30] Citeau, J., Finaud, L., Cammas, J. P. and Demarcq, H. (1989) Questions relative to ITCZ migrations over the tropical Atlantic Ocean, sea surface temperature and Senegal River runoff. Meteorog. Atmos. Phys., 41, 181–190. 10.1007/BF01026109.

[ref31] Clarke, K. R., Gorley, R. N., Somerfield, P. J. and Warwick, R. M. (2014) Change in marine communities: an approach to statistical analysis and interpretation (3rd edn). PRIMER-E Ltd., Plymouth, UK, pp. 262.

[ref32] Clarke, K. R., Somerfield, P. J. and Gorley, R. N. (2008) Testing of null hypotheses in exploratory community analyses: similarity profiles and biota-environment linkage. J. Exp. Mar. Biol. Ecol., 366, 56–69. 10.1016/j.jembe.2008.07.009.

[ref33] Cropper, T. E., Hanna, E. and Bigg, G. R. (2014) Spatial and temporal seasonal trends in coastal upwelling off Northwest Africa, 1981–2012. Deep. Sea. Res. 1 Oceanogr. Res. Pap., 86, 94–111. 10.1016/j.dsr.2014.01.007.

[ref34] Cruz, J., Garrido, S., Pimentel, M. S., Rosa, R., Santos, A. M. P. and Ré, P. (2013) Reproduction and respiration of a climate change indicator species: effect of temperature and variable food in the copepod *Centropages chierchiae*. J. Plankton Res., 35, 1046–1058. 10.1093/plankt/fbt057.

[ref35] Dandonneau, Y. (1973) Étude du phytoplancton sur le plateau continental de Côte d’Ivoire. III—facteurs dynamiques et variations spatio-temporelles. Cah. ORSTOM, sér. Océanogr., 11, 431–454.

[ref36] Dessier, A. (1979) Ecologie, dynamique des peuplements zooplanctoniques côtiers, et plus particulièrement des copépodes, du Sud du golfe de Guinée (côtes du Congo, du Gabon et de L’angola). pp. 389. PhD thesis. Pierre and Marie Curie University, Paris, France.

[ref37] Djakouré, S., Amouin, J., Kouadio, K. Y. and Kacou, M. (2024b) Mesoscale convective systems and extreme precipitation on the west African coast linked to ocean–atmosphere conditions during the monsoon period in the Gulf of Guinea. Atmosphere (Basel)., 15, 194. 10.3390/atmos15020194.

[ref38] Djakouré, S., Konaté, Y., Koné, V., Bosson, K., Koné, M. and Kouadio, K. Y. (2024a) Relationship between the Guinea current and the coastal upwelling in northern of gulf of Guinea. Open J. Mar. Sci., 14, 63–77. 10.4236/ojms.2024.144004.

[ref39] Djakouré, S., Penven, P., Bourlès, B., Koné, V. and Veitch, J. (2017) Respective roles of the Guinea current and local winds on the coastal upwelling in the northern gulf of Guinea. J. Phys. Oceanogr., 47, 1367–1387. 10.1175/JPO-D-16-0126.1.

[ref40] Efon, E., Ngongang, R. D., Meukaleuni, C., Wandjie, B. B. S., Zebaze, S., Lenouo, A. and Valipour, M. (2023) Monthly, seasonal, and annual variations of precipitation and runoff over west and Central Africa using remote sensing and climate reanalysis. Earth Syst. Environ., 7, 67–82. 10.1007/s41748-022-00326-w.

[ref41] Escribano, R., Hidalgo, P., González, H., Giesecke, R., Riquelme-Bugueño, R. and Manríquez, K. (2007) Seasonal and inter-annual variation of mesozooplankton in the coastal upwelling zone off central-southern Chile. Prog. Oceanogr., 75, 470–485. 10.1016/j.pocean.2007.08.027.

[ref42] Fernández-Urruzola, I., Osma, N., Packard, T. T., Gómez, M. and Postel, L. (2014) Distribution of zooplankton biomass and potential metabolic activities across the northern Benguela upwelling system. J. Mar. Syst., 140, 138–149. 10.1016/j.jmarsys.2014.05.009.

[ref43] Finley, A., Banerjee, S., Hjelle, Ø. and Bivand, R. (2024) MBA: Multilevel B-Spline Approximation. R package (Version 0.1-2). Comprehensive R Archive Network (CRAN), Vienna, Austria. https://CRAN.R-project.org/package=MBA.

[ref44] Garcia, T. M., Santos, N. M. O., Campos, C. C., Costa, G. A. S., Belmonte, G., Rossi, S. and Soares, M. O. (2021) Plankton net mesh size influences the resultant diversity and abundance estimates of copepods in tropical oligotrophic ecosystems. Estuar. Coast. Shelf Sci., 249, 107083. 10.1016/j.ecss.2020.107083.

[ref45] Goliat, Y., Ettahiri, O., Baibai, T., Rharbi, N. and Isari, S. (2025) Spatio-temporal variability of mesozooplankton distribution along the canary current large marine ecosystem: a regional perspective. J. Plankton Res., 47, fbae079. 10.1093/plankt/fbae079. 39886552 PMC11781819

[ref46] Greer, A. T., Cowen, R. K., Guigand, C. M., Hare, J. A. and Tang, D. (2014) The role of internal waves in larval fish interactions with potential predators and prey. Prog. Oceanogr., 127, 47–61. 10.1016/j.pocean.2014.05.010.

[ref47] Grigoratou, M., Menden-deuer, S., Mcquatters-gollop, A., Arhonditsis, G., Artigas, L. F., Ayata, S., Bediko, D., Beisner, B. E. et al. (2025) The immeasurable value of plankton to humanity. Bioscience, 75, 706–721. 10.1093/biosci/biaf049. 40919339 PMC12412299

[ref48] Grigoratou, M., Montes, E., Richardson, A. J., Everett, J. D., Acevedo-Trejos, E., Anderson, C., Chen, B., Guy-Haim, T. et al. (2022) The marine biodiversity observation network plankton workshops: plankton ecosystem function, biodiversity, and forecasting—research requirements and applications. Limnol. Oceanogr. Bull., 31, 22–26. 10.1002/lob.10479.

[ref49] Gu, G. and Adler, R. F. (2004) Seasonal evolution and variability associated with the west African monsoon system. J. Clim., 17, 3364–3377. 10.1175/1520-0442(2004)017<3364:SEAVAW>2.0.CO;2.

[ref50] Hardman-Mountford, N. J. and McGlade, J. M. (2002a) Defining ecosystem structure from natural variability: application of principal components analysis to remotely sensed SST. Large Mar. Ecosyst., 11, 67–82. 10.1016/S1570-0461(02)80028-3.

[ref51] Hardman-Mountford, N. J. and McGlade, J. M. (2002b) Variability of physical environmental processes in the Gulf of Guinea and implications for fisheries recruitment. An investigation using remotely sensed SST. Large Mar. Ecosyst., 11, 49–66. 10.1016/S1570-0461(02)80027-1.

[ref52] Hernández Ruiz, L., Ekumah, B., Asiedu, D. A., Albani, G., Acheampong, E., Jónasdóttir, S. H., Koski, M. and Nielsen, T. G. (2021) Climate change and oil pollution: a dangerous cocktail for tropical zooplankton. Aquat. Toxicol., 231, 105718. 10.1016/j.aquatox.2020.105718. 33360235

[ref53] Hopcroft, R. R., Roff, J. C. and Chavez, F. P. (2001) Size paradigms in copepod communities: a re-examination. Hydrobiologia, 453-454, 133–141. 10.1023/A:1013167917679.

[ref54] Höring, F., Cornils, A., Auel, H., Bode, M. and Held, C. (2017) Population genetic structure of *Calanoides natalis* (Copepoda, Calanoida) in the eastern Atlantic Ocean and Benguela upwelling system. J. Plankton Res., 39, 618–630. 10.1093/plankt/fbx035.

[ref55] Houghton, R. W. and Mensah, M. A. (1978) Physical aspects and biological consequences of Ghanaian coastal upwelling. In Boje, R., T. (ed), Upwelling Ecosystems. Springer Berlin Heidelberg, pp. 167–180. 10.1007/978-3-642-66985-9_14

[ref56] Huggett, J., Verheye, H., Escribano, R. and Fairweather, T. (2009) Copepod biomass, size composition and production in the southern Benguela: spatio-temporal patterns of variation, and comparison with other eastern boundary upwelling systems. Prog. Oceanogr., 83, 197–207. 10.1016/j.pocean.2009.07.048.

[ref57] Isari, S., Zervoudaki, S., Calbet, A., Saiz, E., Ptacnikova, R., Nejstgaard, J. C., Sousoni, D., Berger, S. A. et al. (2014) Light-induced changes on the feeding behaviour of the calanoid copepod *Clausocalanus furcatus* (Brady, 1883): evidence from a mesocosm study. J. Plankton Res., 36, 1233–1246. 10.1093/plankt/fbu054.

[ref58] John, A. W. G., Reid, P. C., Batten, S. D. and Anang, E. R. (2002) Monitoring levels of ‘phytoplankton colour’ in the gulf of Guinea using ships of opportunity. Large Mar. Ecosyst., 11, 141–146. 10.1016/S1570-0461(02)80033-7.

[ref59] Kasapidis, P., Siokou, I., Khelifi-Touhami, M., Mazzocchi, M. G., Matthaiaki, M., Christou, E., Fernandez De Puelles, M. L., Gubanova, A. et al. (2018) Revising the taxonomic status and distribution of the *Paracalanus parvus* species complex (Copepoda, Calanoida) in the Mediterranean and black seas through an integrated analysis of morphology and molecular taxonomy. J. Plankton Res., 40, 595–605. 10.1093/plankt/fby036.

[ref60] Khelifi-Touhami, M. and Ounissi, M. (2023) *Paracalanus* Boeck, 1864. ICES Identif. Leafl. Plankton, 199, 1–22. 10.17895/ices.pub.21724394.

[ref61] Koné, M., Djakouré, S., Adon, M., Ta, S. and Kouadio, Y. (2022) Marine heatwaves, upwelling, and atmospheric conditions during the monsoon period at the northern coast of the Gulf of Guinea. Climate, 10, 199. 10.3390/cli10120199.

[ref62] Koranteng, K. A. (2002a) Fish species assemblages on the continental shelf and upper slope off Ghana. Large Mar. Ecosyst., 11, 173–187. 10.1016/S1570-0461(02)80036-2.

[ref63] Koranteng, K. A. (2002b) Status of demersal fishery resources on the inner continental shelf off Ghana. Large Mar. Ecosyst., 11, 261–274. 10.1016/S1570-0461(02)80041-6.

[ref64] Le Borgne, R. and Binet, D. (1979) Dix ans de mesures de biomasses de zooplancton à la station côtière d’Abidjan, 1969–1979. Doc. Scient. Centre Rech, Océanogr. Abidjan, 10, 65–176.

[ref65] Lee, S., Wolberg, G. and Shin, S. Y. (1997) Scattered data interpolation with multilevel B-splines. IEEE Trans. Vis. Comput. Graph., 3, 228–244. 10.1109/2945.620490.

[ref66] Legendre, P. and Anderson, M. J. (1999) Distance-based redundancy analysis: testing multispecies responses in multifactorial ecological experiments. Ecol. Monogr., 69, 1–24. 10.1890/0012-9615(1999)069[0001:DBRATM]2.0.CO;2.

[ref67] Lindley, J. A. and Daykin, S. (2005) Variations in the distributions of *Centropages chierchiae* and *Temora stylifera* (Copepoda: Calanoida) in the north-eastern Atlantic Ocean and western European shelf waters. ICES J. Mar. Sci., 62, 869–877. 10.1016/j.icesjms.2005.02.009.

[ref68] Martin, B., Eggert, A., Koppelmann, R., Diekmann, R., Mohrholz, V. and Schmidt, M. (2015) Spatio-temporal variability of zooplankton biomass and environmental control in the northern Benguela upwelling system: field investigations and model simulation. Mar. Ecol., 36, 637–658. 10.1111/maec.12173.

[ref69] Mazzocchi, M. G. (2020) *Clausocalanus* Giesbrecht, 1888. ICES Identif. Leafl. Plankton, 189, 1–19. 10.17895/ices.pub.5464.

[ref70] Mazzocchi, M. G. and Paffenhöfer, G. A. (1998) First observations on the biology of *Clausocalanus furcatus* (Copepoda, Calanoida). J. Plankton Res., 20, 331–342. 10.1093/plankt/20.2.331.

[ref71] Mazzocchi, M. G. and Paffenhöfer, G. A. (1999) Swimming and feeding behaviour of the planktonic copepod *Clausocalanus furcatus*. J. Plankton Res., 21, 1501–1518. 10.1093/plankt/21.8.1501.

[ref72] Mensah, A. (1995) The occurence of zooplankton off Tema during the period 1969-1992. In Bard, F. X. and Koranteng, K. A. (eds.), Dynamics and Use of Sardinella Resources from Upwelling off Ghana and Ivory Coast, ORSTOM Editions, Paris, pp. 279–289.

[ref73] Mensah, M. A. (1974) The occurrence of the marine copepod *Calanoides carinatus* (Krøyer) in Ghanaian waters. Ghana J. Sci., 14, 147–166.

[ref74] Mensah, M. A. (1991) The influence of climatic changes on the coastal oceanography of Ghana. In Cury, P. and Roy, C. (eds.), Pêcheries Ouest-Africaines: variabilité, instabilité et Changement, ORSTOM, Paris, pp. 67–79.

[ref75] Mensah, M. A. and Quaatey, S. N. K. (2002) An overview of the fishery resources and fishery research in the gulf of Guinea. Large Mar. Ecosyst., 11, 227–240. 10.1016/S1570-0461(02)80039-8.

[ref76] Miloslavich, P., Bax, N. J., Simmons, S. E., Klein, E., Appeltans, W., Aburto-Oropeza, O., Andersen Garcia, M., Batten, S. D. et al. (2018) Essential ocean variables for global sustained observations of biodiversity and ecosystem changes. Glob. Chang. Biol., 24, 2416–2433. 10.1111/gcb.14108. 29623683

[ref77] Ndour, I., Berraho, A., Fall, M., Ettahiri, O. and Sambe, B. (2018) Composition, distribution and abundance of zooplankton and ichthyoplankton along the Senegal-Guinea maritime zone (West Africa). Egypt. J. Aquat. Res., 44, 109–124. 10.1016/j.ejar.2018.04.001.

[ref78] Nguyen, K. D. T., Morley, S. A., Lai, C. H., Clark, M. S., Tan, K. S., Bates, A. E. and Peck, L. S. (2011) Upper temperature limits of tropical marine ectotherms: global warming implications. PLoS One, 6, 6–13. 10.1371/journal.pone.0029340. PMC3248430 22242115

[ref79] Nieto, K. and Mélin, F. (2017) Variability of chlorophyll-*a* concentration in the Gulf of Guinea and its relation to physical oceanographic variables. Prog. Oceanogr., 151, 97–115. 10.1016/j.pocean.2016.11.009. 28298724 PMC5339419

[ref80] Oksanen, J., Simpson, G., Blanchet, F., Kindt, R., Legendre, P., Minchin, P., O’Hara, R., Solymos, P. et al. (2025) Vegan: community ecology package. R package (version 2.6-10). Comprehensive R Archive Network (CRAN), Vienna, Austria. https://cran.r-project.org/package=vegan.

[ref81] Omori, M. and Ikeda, T. (1984) Methods in Marine Zooplankton Ecology, John Wiley and Sons, New York.

[ref82] Peralba, À., Mazzocchi, M. G. and Harris, R. P. (2017) Niche separation and reproduction of *Clausocalanus* species (Copepoda, Calanoida) in the Atlantic Ocean. Prog. Oceanogr., 158, 185–202. 10.1016/j.pocean.2016.08.002.

[ref83] Peters, J., Albaina, A., Blanco-Bercial, L., Bucklin, A., Di Capua, I., Dos Santos, A., Falkenhaug, T., Fernandez de Puelles, M. et al. (2025) Taxonomic uncertainty in North Atlantic and Mediterranean zooplankton limits species-level monitoring accuracy. ICES J. Mar. Sci., 82, fsaf077. 10.1093/icesjms/fsaf077.

[ref84] Pezennec, O. and Koranteng, K. A. (1998) Changes in the dynamics and biology of small pelagic fisheries off cote-d’Ivoire and Ghana: an ecological puzzle. In Durand, M.-H., Cury, P., Mendelssohn, R., Roy, C., Bakun, A., and Pauly, D. (eds.), Global Versus Local Changes in Upwelling Systems,ORSTOM Editions, Paris, pp. 329–343.

[ref85] Picaut, J. (1983) Propagation of the seasonal upwelling in the eastern equatorial Atlantic. J. Phys. Oceanogr., 13, 18–37. 10.1175/1520-0485(1983)013<0018:POTSUI>2.0.CO;2.

[ref86] Pielou, E. C. (1966) Species-diversity and pattern-diversity in the study of ecological succession. J. Theor. Biol., 10, 370–383. 10.1016/0022-5193(66)90133-0. 5964400

[ref87] Postel L, Fock, H, & Hagen W (2000). Biomass and abundance. In: Harris R. et al. (eds.), ICES Zooplankton Methodology Manual. Academic Press, London, pp. 83–192. 10.1016/B978-0-12-327645-2.X5000-2

[ref88] R Core Team (2024) R: A Language and Environment for Statistical Computing (Version 4.3.3). R Foundation for Statistical Computing, Vienna, Austria. https://www.R-project.org/.

[ref89] Ratnarajah, L., Abu-Alhaija, R., Atkinson, A., Batten, S., Bax, N. J., Bernard, K. S., Canonico, G., Cornils, A. et al. (2023) Monitoring and modelling marine zooplankton in a changing climate. Nat. Commun., 14, 564. 10.1038/s41467-023-36241-5. 36732509 PMC9895051

[ref90] Richardson, A. J., Davies, C., Slotwinski, A., Coman, F., Tonks, M., Rochester, W., Murphy, N., Beard, J. et al. (2013) Australian Marine Zooplankton: Taxonomic Sheets. pp. 294. Institute for Marine and Antarctic Studies, University of Tasmania, Hobart, Tasmania, Australia.

[ref91] Rose, M. (1933) Faune de France: Copépodes pélagiques, Vol. 26, Lechevalier, Paris, p. pp377.

[ref92] Roy, C. (1995) The Côte d’ Ivoire and Ghana Coastal upwellings: Dynamics and changes. In Bard, F. X. and Koranteng, K. A. (eds.), Dynamics and Use of Sardinella Resources from Upwelling off Ghana and Ivory Coast, ORSTOM Editions, Paris. pp. 346–361.

[ref93] RStudio Team (2024) RStudio: Integrated Development for R (Version 2023.6.2.561), http://www.rstudio.com/.

[ref94] Satia, B. P. (2016) An overview of the large marine ecosystem programs at work in Africa today. Environ. Dev., 17, 11–19. 10.1016/j.envdev.2015.06.007.

[ref95] Shannon, C. E. and Weaver, W. (1949) The Mathematical Theory of Communication, The university of Illinois Press, Urbana, p. 117.

[ref96] Sobrinho-Gonçalves, L., Moita, M. T., Garrido, S. and Cunha, M. E. (2013) Environmental forcing on the interactions of plankton communities across a continental shelf in the eastern Atlantic upwelling system. Hydrobiologia, 713, 167–182. 10.1007/s10750-013-1500-2.

[ref97] Sultan, B., Janicot, S. and Drobinski, P. (2007) Characterization of the diurnal cycle of the west African monsoon around the monsoon onset. J. Clim., 20, 4014–4032. 10.1175/JCLI4218.1.

[ref98] Teuber, L., Hagen, W., Bode, M. and Auel, H. (2019) Who is who in the tropical Atlantic? Functional traits, ecophysiological adaptations and life strategies in tropical calanoid copepods. Prog. Oceanogr., 171, 128–135. 10.1016/j.pocean.2018.12.006.

[ref99] Thiriot, A. (1978) Zooplankton Communities in the West African upwelling area. In Boje, R. and Tomczak, M. (eds), Upwelling Ecosystems. Springer Berlin Heidelberg, Berlin, Heidelberg, pp. 32–61. 10.1007/978-3-642-66985-9_5

[ref100] Trégouboff, G. and Rose, M. (1957) Manuel de Planctonologie Méditerranéenne, Centre National de la Recherche Scientifique, Paris, pp. 218.

[ref101] Vázquez, R., Parras-Berrocal, I. M., Koseki, S., Cabos, W., Sein, D. V. and Izquierdo, A. (2023) Seasonality of coastal upwelling trends in the Mauritania-Senegalese region under RCP8.5 climate change scenario. Sci. Total Environ., 898, 166391. 10.1016/j.scitotenv.2023.166391. 37597551

[ref102] Whitaker, D. and Christman, M. (2014) Clustsig: Significant Cluster Analysis. R Package (Version 1.1). GitHub. clustsig repository

[ref103] Wiafe, G., Dovlo, E. and Agyekum, K. (2016) Comparative productivity and biomass yields of the Guinea current LME. Environ. Dev., 17, 93–104. 10.1016/j.envdev.2015.07.001.

[ref104] Wiafe, G. and Frid, C. (2001) Marine Zooplankton of West Africa. Marine Biodiversity Capacity Building in the West African Sub-region, Darwin Initiative Report 5 (Ref. 162/7/451)

[ref105] Wiafe, G., Yaqub, H. B., Mensah, M. A. and Frid, C. L. J. (2008) Impact of climate change on long-term zooplankton biomass in the upwelling region of the Gulf of Guinea. ICES J. Mar. Sci., 65, 318–324. 10.1093/icesjms/fsn042.

[ref106] Wickham, H. (2016) ggplot2: Elegant Graphics for Data Analysis. R package (Version 3.5.1). Comprehensive R Archive Network (CRAN), Vienna, Austria.

[ref107] Yaqub, H. B. (2000) Copepods in Ghanaian Coastal Waters—Abundance and Diversity. pp. 49. Master Thesis. University of Southampton UK.

[ref108] Zervoudaki, S., Christou, E. D., Nielsen, T. G., Siokou-Frangou, I., Assimakopoulou, G., Giannakourou, A., Maar, M., Pagou, K. et al. (2007) The importance of small-sized copepods in a frontal area of the Aegean Sea. J. Plankton Res., 29, 317–338. 10.1093/plankt/fbm018.

[ref109] Zuur, A. F., Ieno, E. N. and Elphick, C. S. (2010) A protocol for data exploration to avoid common statistical problems. Methods Ecol. Evol., 1, 3–14. 10.1111/j.2041-210X.2009.00001.x.

