# Supplementary material for: Mesozooplankton distribution in the Guinea Current Large Marine Ecosystem (GCLME) during the west African monsoon: a comparison across subsystems
Source: J Plankton Res. 2026 Jul 2;48(4):fbag050. doi: 10.1093/plankt/fbag050 (PMC13326642; doi:10.1093/plankt/fbag050)
Supplement: Anadoli_et_al_supplementary_material_revised_fbag050 [file anadoli_et_al_supplementary_material_revised_fbag050.docx]

**SUPPLEMENATRY MATERIAL**

| **Table S1.** Mean values of abundance and dry weight per subsystem: W-GCLME (Abundance: N=31, Biomass: N=34) and the C- GCLME (Abundance: N=31, Biomass: N=31). Ranges of values are provided in parentheses. Values have been also expressed per m^3^ to allow comparison with other studies. | | | | |
| --- | --- | --- | --- | --- |
|  | **Abundance** | | **Dry weight** | |
|  | ind m^-2^ | ind m^-3^ | g m^-2^ | mg m^-3^ |
| W-GCLME | 73,315  (21,173-240,960) | 1,290  (126-7,092) | 1.9  (0.11-7.02) | 24.66  (2.66-100.75) |
| C-GCLME | 131,488  (26,976-287,520) | 2,058  (212-8,947) | 4.44  (0.32-25.1) | 64.12  (3.18-309.28) |
| All area | 102,402  (21,173-287,520) | 1,674  (126-8,947) | 3.11  (0.11-25.1) | 43.48  (2.66-309.28) |

| **Table S2.** List of total copepod taxa identified in GCLME in summer 2017. Rare taxa detected only during an exhaustive scanning of the entire sample are **bold.** | | | |
| --- | --- | --- | --- |
| **Copepods** | |  | *Labidocera nerii* |
| **Calanoida** |  |  | *Labidocera scotti* |
| Acartidae | *Acartia danae* |  | ***Pontellina platychela*** |
|  | *Acartia negligens* |  | *Pontellopsis brevis* |
| Aetideidae | *Aetideopsis* spp. |  | ***Pontellopsis perspicax*** |
|  | ***Aetideus acutus*** | Pseudodiaptomidae | *Pseudodiaptomus serricaudatus* |
|  | ***Aetideus armatus*** | Rhincalanidae | *Rhincalanus cornutus* |
|  | *Paivella inaceae* | Scolecitrichidae | *Scaphocalanus curtus* |
| Augaptilidae | *Euaugaptilus hecticus* |  | ***Scaphocalanus echinatus*** |
|  | ***Haloptilus acutifrons*** |  | *Scolecithricella abyssalis* |
|  | *Haloptilus longicornis* |  | *Scolecithricella profunda* |
|  | ***Haloptilus ornatus*** |  | *Scolecithrix bradyi* |
| Calanidae | *Calanoides natalis* |  | *Scolecithrix danae* |
|  | *Nanocalanus minor* |  | *Scolecitrichopsis ctenopus* |
|  | *Undinula vulgaris* |  | *Scolecitrichopsis tenuipes* |
| Candaciidae | ***Candacia bipinnata*** |  | *Scottocalanus helenae* |
|  | *Candacia curta* | Subeucalanidae | *Subeucalanus crassus* |
|  | *Candacia pachydactyla* |  | *Subeucalanus monachus* |
|  | ***Candancia varicans*** |  | *Subeucalanus pileatus* |
| Centropagidae | *Centropages bradyi* |  | *Subeucalanus subtenuis* |
|  | *Centropages chierchiae* | Temoridae | *Temora stylifera* |
|  | *Centropages velificatus* |  | *Temora turbinata* |
|  | *Centropages violaceus* | **Cyclopoida** |  |
| Clausocalanidae | *Clausocalanus furcatus* | Corycaeidae | *Agetus flaccus* |
|  | *Clausocalanus mastigophorus* |  | *Agetus limbatus* |
|  | *Clausocalanus parapergens* |  | ***Corycaeus clausi*** |
|  | *Clausocalanus pergens* |  | *Corycaeus speciosus* |
|  | *Ctenocalanus vanus* |  | *Ditrichocorycaeus africanus* |
|  | *Microcalanus pygmaeus* |  | ***Ditrichocorycaeus* sp.** |
| Diaixidae | *Diaixis pygmaea* |  | *Farranula gracilis* |
| Euchaetidae | ***Euchaeta acuta*** |  | *Farranula rostrata* |
|  | *Euchaeta paraconcinna* |  | *Onychocorycaeus giesbrechti* |
|  | ***Paraeuchaeta hebes*** |  | *Onychocorycaeus ovalis* |
| Fosshageniidae | *Temoropia mayumbaensis* |  | *Urocorycaeus furcifer* |
| Heterorhabdidae | *Heterorhabdus guineanensis* | Lubbockiidae | *Lubbockia squillimana* |
|  | ***Heterorhabdus spinifrons*** | Oithonidae | *Oithona nana* |
|  | ***Heterostylites longicornis*** |  | *Oithona plumifera* |
| Lucicutidae | *Lucicutia clausi* | Oncaeidae | *Oncaea-Triconia* spp. |
|  | *Lucicutia flavicornis* | Sapphirinidae | ***Sapphirina angusta*** |
| Metridinidae | *Pleuromamma abdominalis* |  | ***Sapphirina intestinata*** |
|  | *Pleuromamma gracilis* |  | *Sapphirina metallina* |
| Paracalanidae | ***Acrocalanus andersoni*** |  | *Sapphirina nigromagulata* |
|  | ***Acrocalanus longicornis*** |  | *Sapphirina ovatolanceolata* |
|  | ***Calocalanus contractus*** |  | ***Sapphirina scarlata*** |
|  | *Calocalanus pavo* |  | ***Vettoria granulosa*** |
|  | *Calocalanus plumulosus* | **Harpacticoida** |  |
|  | ***Calocalanus tenuis*** | Ectinosomatidae | *Microsetella rosea* |
|  | *Delibus nudus* | Miraciidae | *Distioculus minor* |
|  | ***Paracalanus indicus*** |  | *Macrosetella gracilis* |
|  | ***Paracalanus tropicus*** |  | *Miracia efferata* |
|  | *Parvocalanus scotti* | Peltidiidae | *Clytemnestra-Goniopsyllus* spp. |
|  | *Mecynocera clausi* | Tachididae | *Euterpina acutifrons* |
| Tachididae | *Euterpina acutifrons* |  |  |

**Table S3.** Average values of taxonomic richness (S), Shannon-Weiner index (H) and Pielou’s evenness index (J), for copepod and diplostracan genera across the W-GCLME and C-GCLME, with standard errors in parentheses. ANOVA results show the effect of subsystem (W-GCLME vs. C-GCLME) on each diversity index.

|  | **S** | **H** | **J** |
| --- | --- | --- | --- |
| **Area** |  |  |  |
| W-GCLME | 20.74 (0.890) | 2.20 (0.040) | 0.28 (0.004) |
| C-GCLME | 22.00 (0.940) | 2.30 (0.040) | 0.28 (0.003) |
|  |  |  |  |
| **ANOVA (W-GCLME vs. C-GCLME)** | | | |
| Pr (>F) | 0.336 | 0.281 | 0.877 |
| F | 0.942 | 1.185 | 0.024 |

| **Table S4:** SIMPER analysis results presenting average abundance (ind. m^-2^) and contribution (%) of copepod and diplostracan taxa  responsible for up to 80% of similarity within the station groups defined by the cluster analysis (Group G1: N = 4, Group G2: N = 4,  Group G3: N = 25, Group G4: N = 4, Group G5: N = 25). The standard errors are provided in parentheses. The colour gradient indicates high  and low values of contribution. Absence of taxa and low values of contribution (%) are presented with -. | | | | | | | | | | |
| --- | --- | --- | --- | --- | --- | --- | --- | --- | --- | --- |
|  | **Average abundance** | | | | | **Contribution (%)** | | | | |
| **Taxa** | **G1** | **G2** | **G3** | **G4** | **G5** | **G1** | **G2** | **G3** | **G4** | **G5** |
| *Calanoides natalis* | - | - | 2.62 (1.26) | 73.42 (30.12) | 45.95 (9.08) |  |  |  | **6.35** | **2.16** |
| *Calocalanus* spp. | - | 10.34 (3.5) | 29.85 (3.22) | 18.65 (10.77) | 34.45 (5.04) |  |  | **3.06** |  |  |
| *Centropages chierchiae* | - | 2.34 (2.34) | 0.44 (0.44) | 19.07 (7.31) | 63.72 (8.56) |  |  |  |  | **4.01** |
| *Centropages velificatus* | 36.65 (7.68) | 44.75 (8.21) | 19.79 (3.59) | 8.77 (3.22) | 27.51 (4.14) | **4.99** | **7.04** |  |  |  |
| *Clausocalanus furcatus* | 3.87 (3.87) | 24.61 (2.69) | 54.44 (6.64) | 4.9 (2.83) | 16.16 (3.59) |  | **4.32** | **5.33** |  |  |
| *Clausocalanus* spp. | 10.16 (4.5) | 15.49 (8.94) | 72.18 (8.13) | 22.45 (15.1) | 64.29 (7.05) |  |  | **7.07** |  | **4.28** |
| Corycaeidae | 62.11 (5.97) | 29.35 (6.02) | 36.95 (3.73) | 26.52 (6.55) | 63.05 (4.61) | **10.36** | **4.26** | **3.88** |  | **5.32** |
| *Ctenocalanus vanus* | - | 1.65 (1.65) | 11.3 (2.83) | 28.92 (6.09) | 37.29 (5.66) |  |  |  | **4.15** |  |
| *Ditrichocorycaeus africanus* | 68.77 (20.42) | 15.09 (5.74) | 7.81 (2.06) | 3.46 (3.46) | 41.97 (5.15) | **8.34** |  |  |  | **3.05** |
| *Euchaeta paraconcinna* | 3.87 (3.87) | 19.17 (7.41) | 46.56 (4.02) | 18.12 (7.38) | 25.38 (3.51) |  |  | **5.06** |  |  |
| *Euterpina acutifrons* | 60.83 (15.92) | 21.68 (12.94) | 16.17 (4.31) | 41.35 (3.76) | 78.98 (8.89) | **7.99** |  |  | **7.06** | **5.52** |
| *Farranula gracilis* | 3.16 (3.16) | 18.33 (2.74) | 34.71 (4.91) | 2.45 (2.45) | 15.14 (4.04) |  |  | **3.06** |  |  |
| *Macrosetella gracilis* | 8.94 (5.42) | 23.05 (4) | 32.19 (2.86) | - | 9.37 (2.62) |  | **3.72** | **3.58** |  |  |
| *Oithona nana* | 56.77 (11.21) | 51.24 (18.35) | 36.85 (2.64) | 42.8 (6.74) | 37.64 (4.65) | **8.02** | **5.54** | **4.32** | **6.49** | **2.55** |
| *Oithona plumifera* | 3.16 (3.16) | 18.13 (2.23) | 40.72 (3.35) | 30.04 (5.7) | 48.22 (5.49) |  | **3.1** | **4.49** | **4.27** | **3.36** |
| *Oithona* spp. | 17.43 (7.84) | 26.04 (6.09) | 54.88 (4.72) | 58.3 (14.97) | 93.47 (7.34) |  | **3.75** | **6.04** | **7.34** | **7.22** |
| *Oncaea-Triconia* spp. | 21.19 (8.77) | 38.67 (3.5) | 93.28 (6.65) | 75.76 (8.65) | 127.97 (7.54) |  | **7.05** | **10.84** | **12.79** | **10.69** |
| *Onychocorycaeus giesbrechti* | 37.98 (3.33) | 22.61 (4.34) | 22.09 (2) | 30.11 (3.73) | 43.25 (4.14) | **6.41** | **3.39** | **2.43** | **4.84** | **3.2** |
| *Paracalanus parvus* complex | 58.34 (5.53) | 37.72 (6.48) | 48.67 (2.84) | 31.99 (11.78) | 70.25 (5.15) | **9.65** | **6.13** | **5.97** | **3.72** | **5.66** |
| *Paracalanus* spp. | 97.55 (15.44) | 59.85 (7.03) | 80.99 (5.59) | 81.93 (12.53) | 115.11 (7.33) | **14.88** | **10.32** | **9.73** | **12.58** | **9.67** |
| *Parvocalanus scotti* | - | 89 (41.34) | 8.43 (3.43) | 4.9 (4.9) | 33.19 (7.34) |  | **6.66** |  |  |  |
| *Penilia avirostris* | 26.38 (14.65) | - | - | 2.45 (2.45) | 50.16 (7.31) |  |  |  |  | **3** |
| *Subeucalanus* spp. | 46.15 (14.9) | 41.07 (4.54) | 38.11 (4.86) | 65.1 (23.76) | 71.81 (8.26) |  | **7.2** | **3.58** | **6.48** | **4.92** |
| *Temora* spp. | 83.7 (15.98) | 26.07 (12.12) | 20.62 (3.33) | 34.01 (4.45) | 70.45 (6.8) | **11.86** |  |  | **5.39** | **5.5** |
| *Temora stylifera* | 18.29 (7.42) | 29.65 (5.18) | 18.68 (2.85) | 14.67 (6.55) | 24.85 (3.18) |  | **4.79** |  |  |  |
| *Undinula vulgaris* | - | 22.99 (4.58) | 26.86 (3.5) | 3.87 (3.87) | 6.51 (2.63) |  | **3.5** | **2.37** |  |  |

| **Table S5**. Results of the distance-based redundancy analysis (dbRDA) showing the contribution of environmental predictors to the variation in the copepod and diplostracan assemblage structure | | | | |
| --- | --- | --- | --- | --- |
| **Predictors** | **Sum of squares** | **F-statistic** | **Pr (>F)** | **Variation explained (%)** |
| Temperature | 1 | 11.36 | 0.001 | 13.76 |
| Sampling depth | 0.69 | 7.76 | 0.001 | 9.40 |
| Chlorophyll-a | 0.3 | 3.39 | 0.004 | 4.11 |
| Salinity | 0.27 | 3.03 | 0.005 | 3.67 |

| **Table S6.** Model selection output using the forward selection procedure and the Akaike Criterion (AIC). Predictors listed in order of contribution to the total explained variation (statistically significant p>0.01 are in bold) | | | |
| --- | --- | --- | --- |
| **Predictors** | **AIC** | **F** | **Pr(>F)** |
| + Temperature | 116.96 | 9.57 | **0.005** |
| + Sampling depth | 111.81 | 7.22 | **0.005** |
| + Salinity | 110.40 | 3.28 | **0.005** |
| + Chlorophyll-a | 109.19 | 3.03 | **0.005** |
|  |  |  |  |


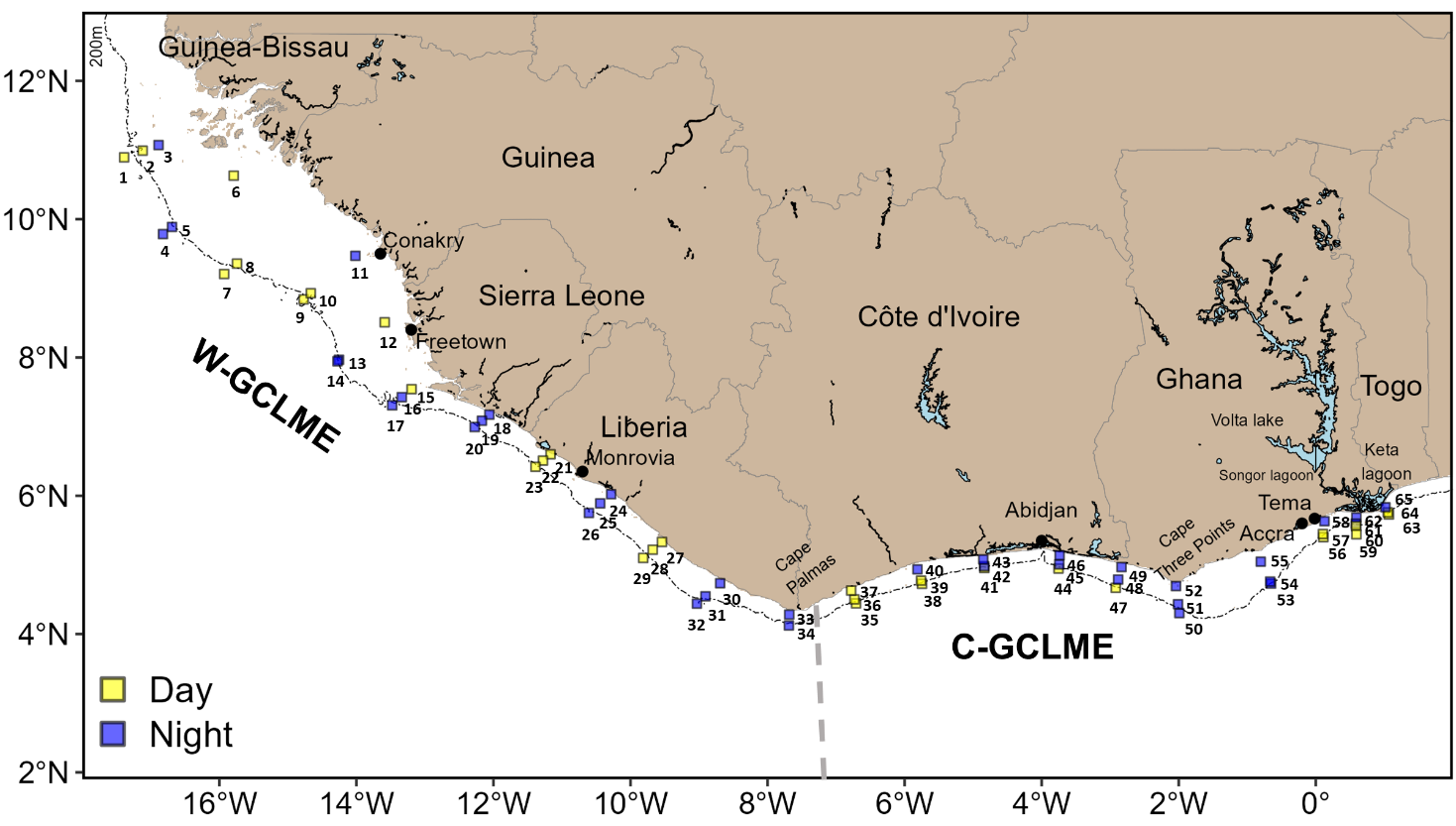


**Figure S1**: Map of the mesozooplankton sampling stations in the Western (W-GCLME) and Central (C-GCLME) Guinea Current Large Marine Ecosystem, indicating the time of sampling (Day and Night) at each station. W-GCLME: 15 stations sampled during day, 19 at night; C-GCLME: 13 during the day, 18 at night.


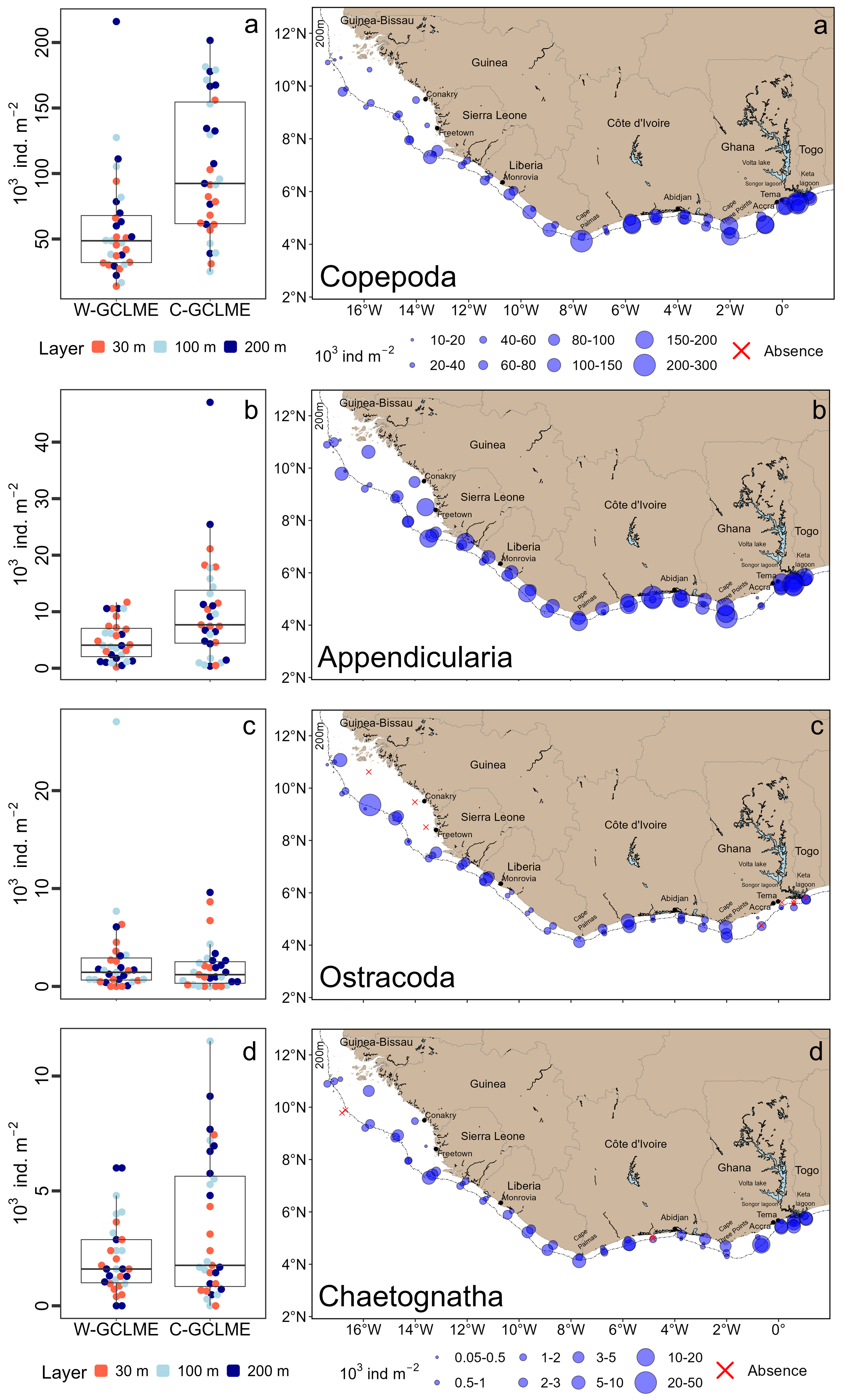


**Figure S2:** Boxplots and spatial distribution of abundance (individuals m^-2^) of major mesozooplankton groups. Copepoda (a), Appendicularia (b), Ostracoda (c) and Chaetognatha (d) for the Western (W-GCLME) and Central (C-GCLME) Guinea Current Large Marine Ecosystem and for the different sampling layers. Scale bars are different for Copepoda and the rest of the major groups


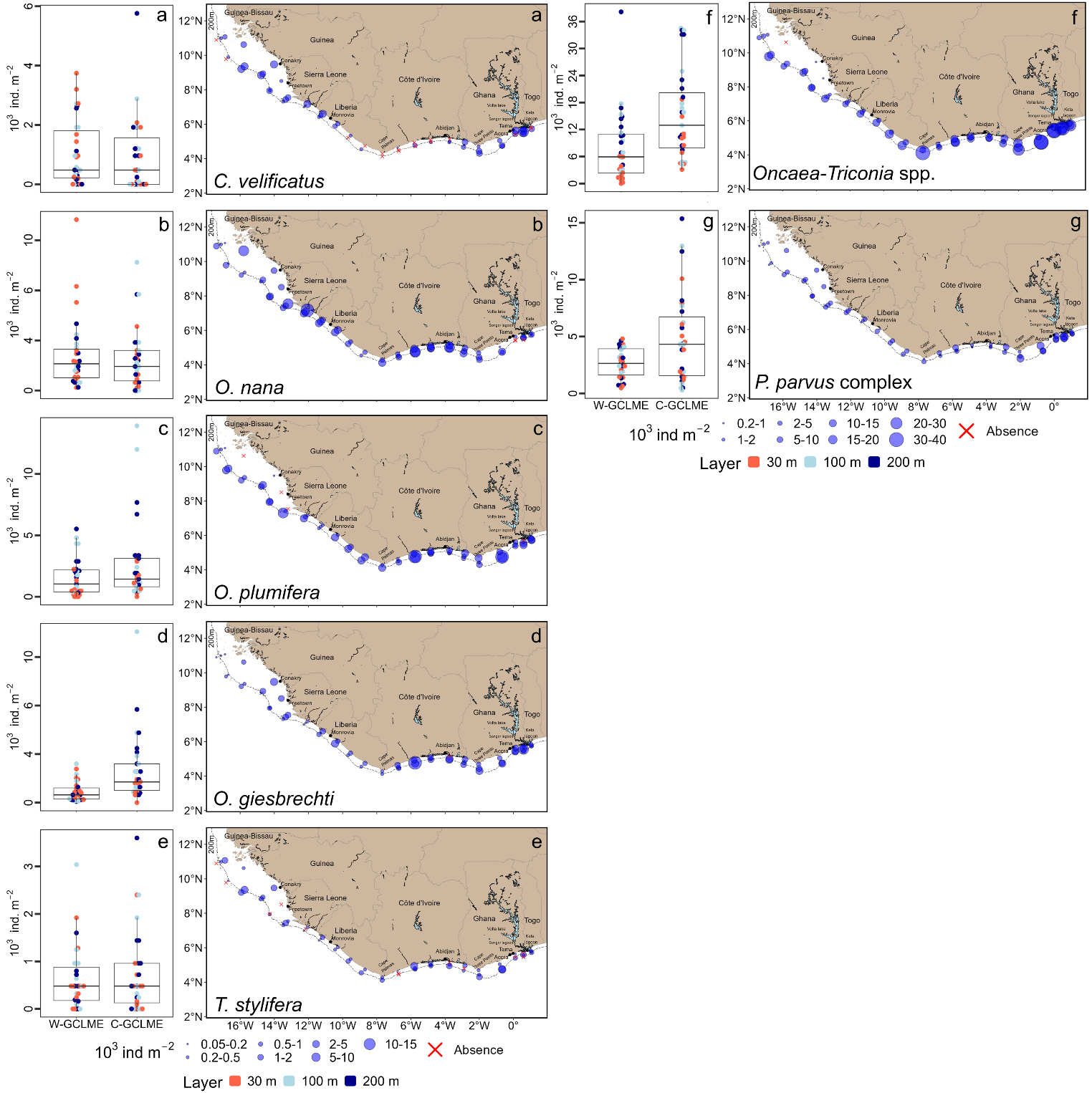


**Figure S3:** Boxplots and spatial distribution of abundance (individuals m^-2^) of upbiquitous taxa. *Centropages velificatus* (a), *Oithona nana* (b), *Oithona plumefera* (c), *Onychocorycaeus giesbrechti* (d) and *Temora stylifera* (e), *Oncaea-Triconia* spp. (f) and *Paracalanus parvus* complex (g) for the Western (W-GCLME) and Central (C-GCLME) Guinea Current Large Marine Ecosystem and for the different sampling layers. Note: Scale bars apply column wise.


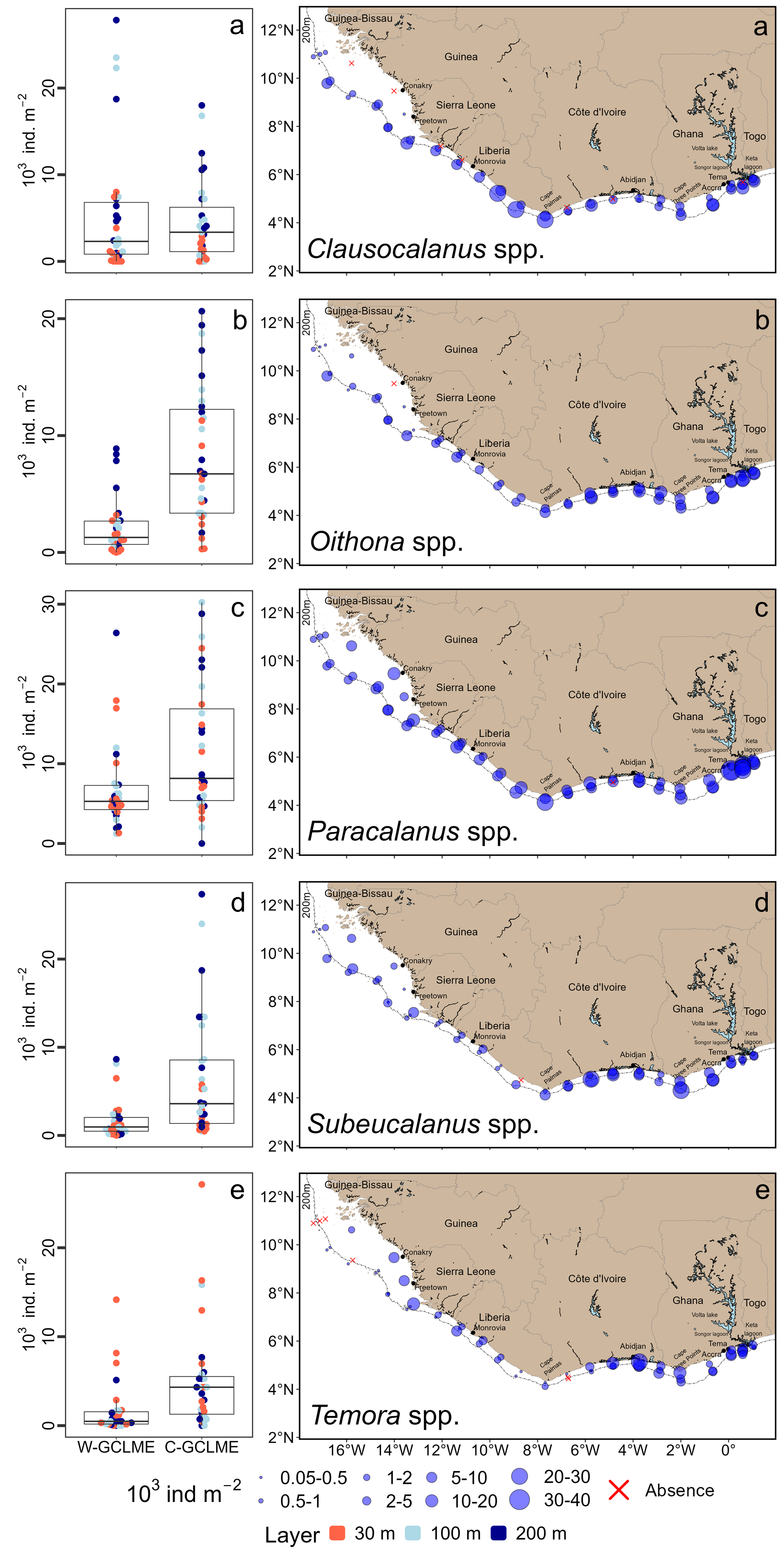


**Figure S4:** Boxplots and spatial distribution of abundance (individuals m^-2^) of the copepodites of the genera *Clausocalanus* (a), *Oithona* (b), *Paracalanus* (c), *Subeucalanus* (d) and *Temora* (e) for the Western (W-GCLME) and Central (C-GCLME) Guinea Current Large Marine Ecosystem and for the different sampling layers.


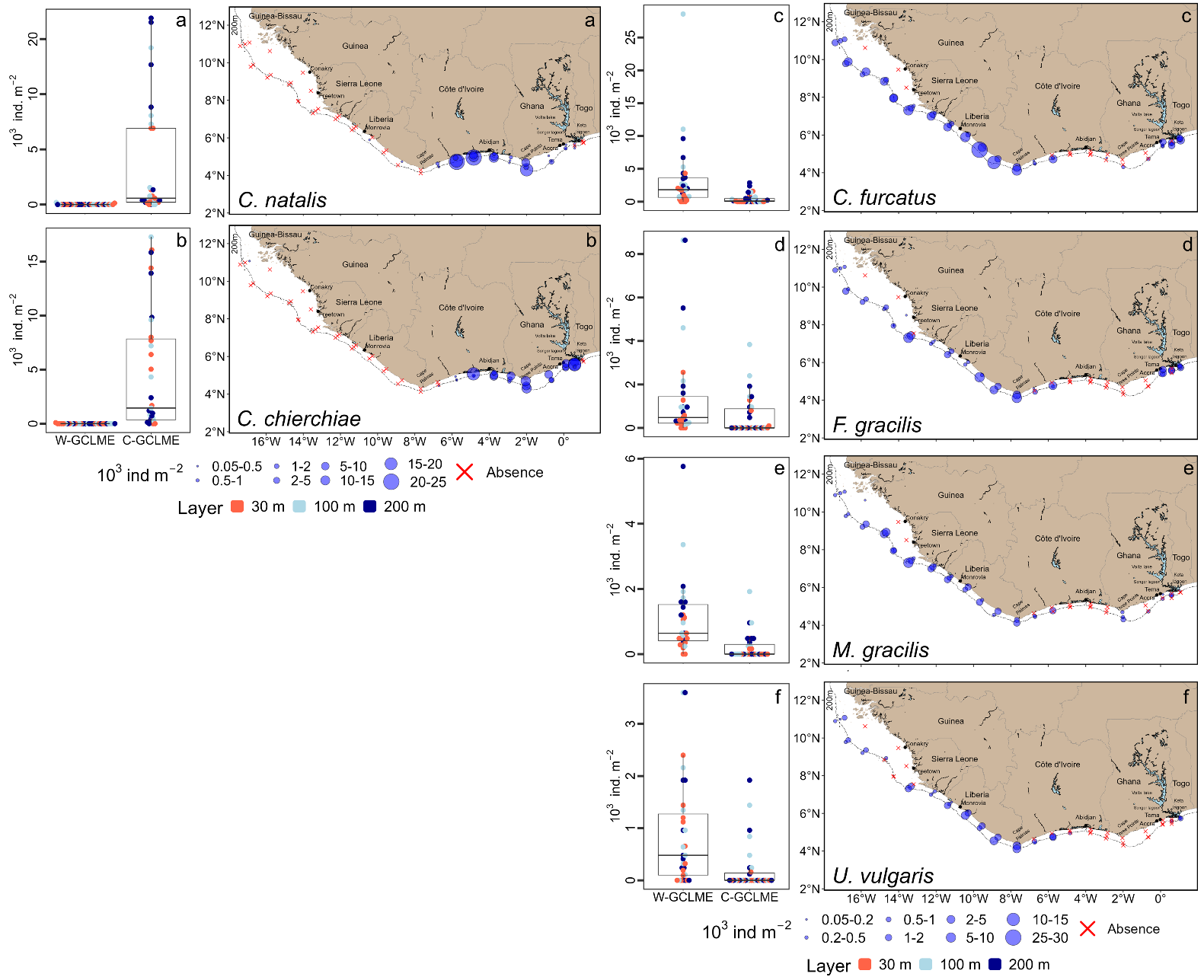


**Figure S5:** Boxplots and spatial distribution of abundance (individuals m^-2^) of *Calanoides natalis* (a), *Centropages chierchiae* (b) *Clausocalanus furcatus* (c), *Faranula gracilis* (d), *Macrosetella gracilis* (e), and *Undinula vulgaris* (f) for the Western (W-GCLME) and Central (C-GCLME) Guinea Current Large Marine Ecosystem and for the different sampling layers. Note: Scale bars apply column wise.


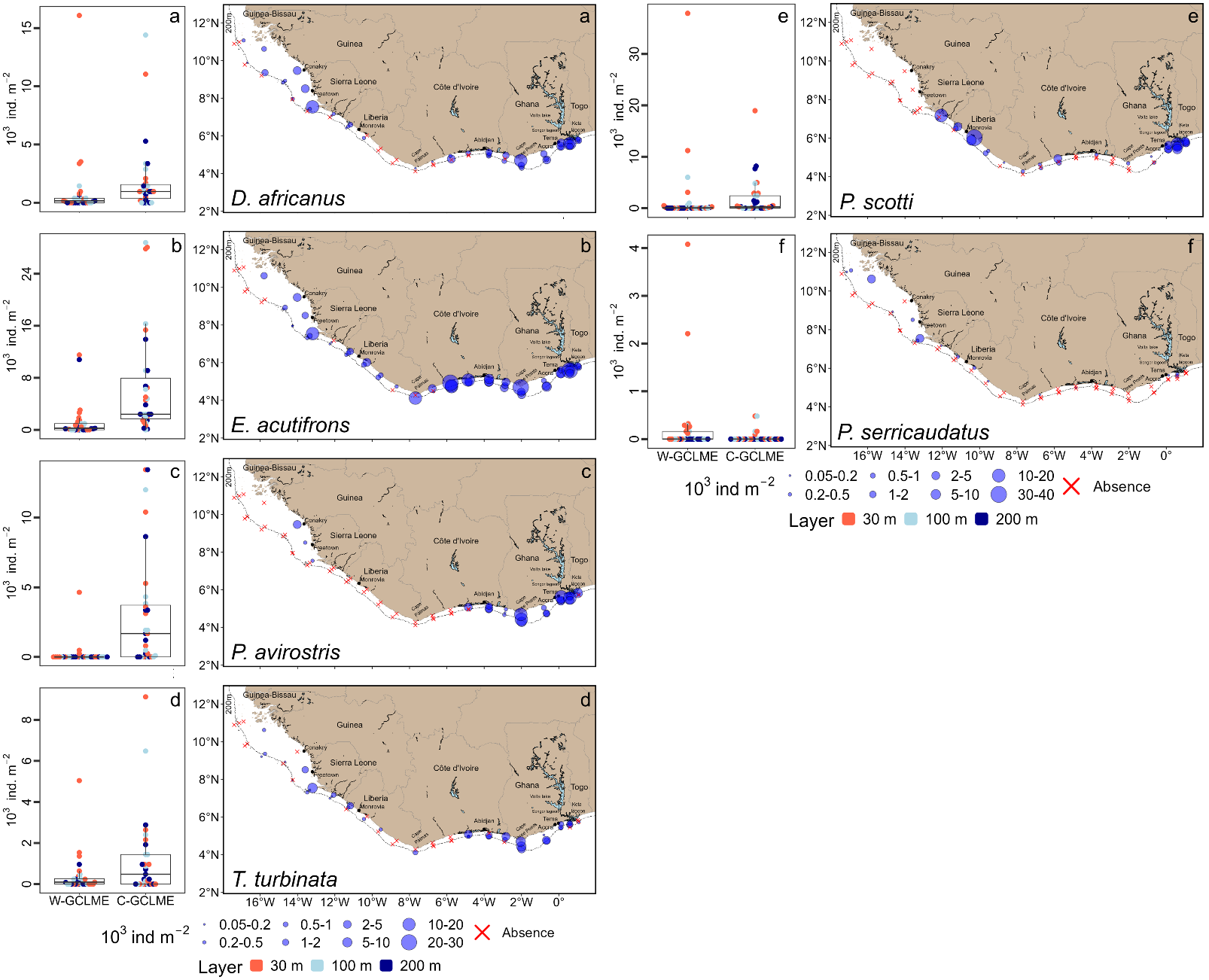


**Figure S6:** Boxplots and spatial distribution of abundance (individuals m-2) of *Ditrichocorycaeus africanus* (a), *Euterpina acutifrons* (b), *Penilia avirostris* (c)*,* *Temora turbinata* (d) *Parvocalanus scotti* (e) and *Pseudodiaptomus serricaudatus* (f) for the Western (W-GCLME) and Central (C-GCLME) Guinea Current Large Marine Ecosystem and for the different sampling layers. Note: Scale bars apply column wise.

**
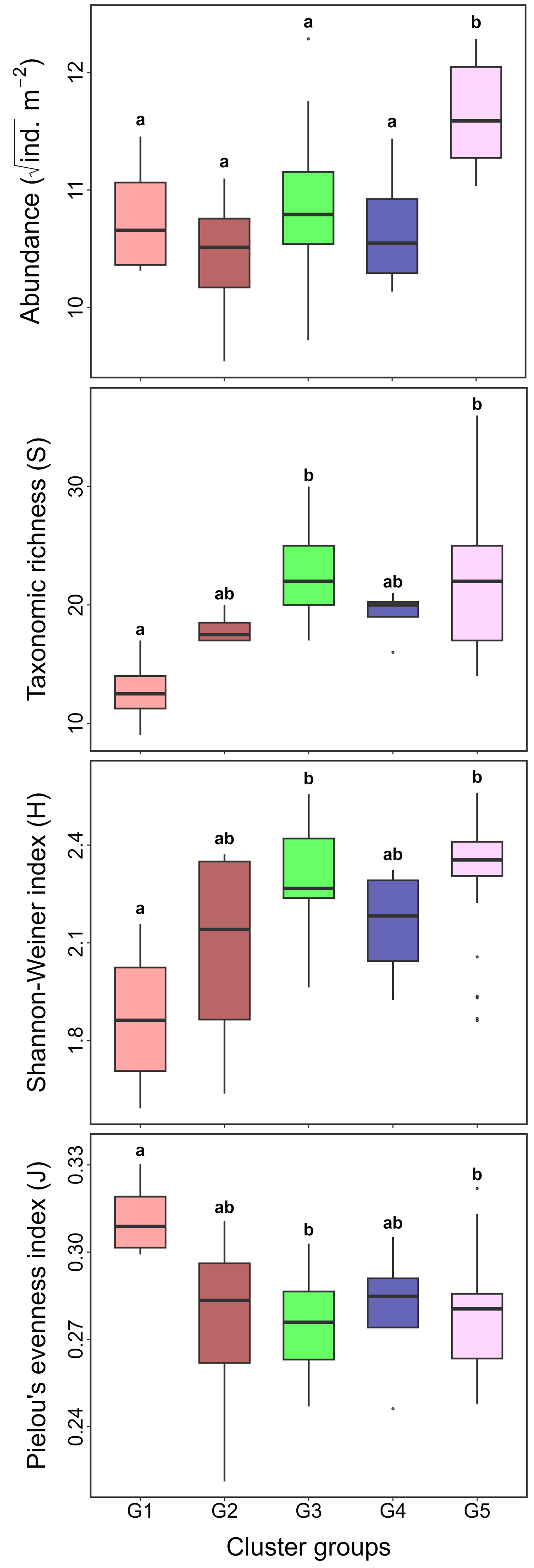
**

**Figure S7:** Box-plots showing total abundance, taxonomic richness (S), Shannon-Weiner index (H) and Pielou’s evenness index (J), for copepod and diplostracan genera across the five cluster groups (G1-5). The size of the box-plots is determined by the upper and lower quartile, with the median indicated by a horizontal black line within the box. Outliers are represented by black dots outside the box. Tukey's HSD test output is presented above the boxplots as letters (different letters indicate statistically significant differences among cluster groups)

**
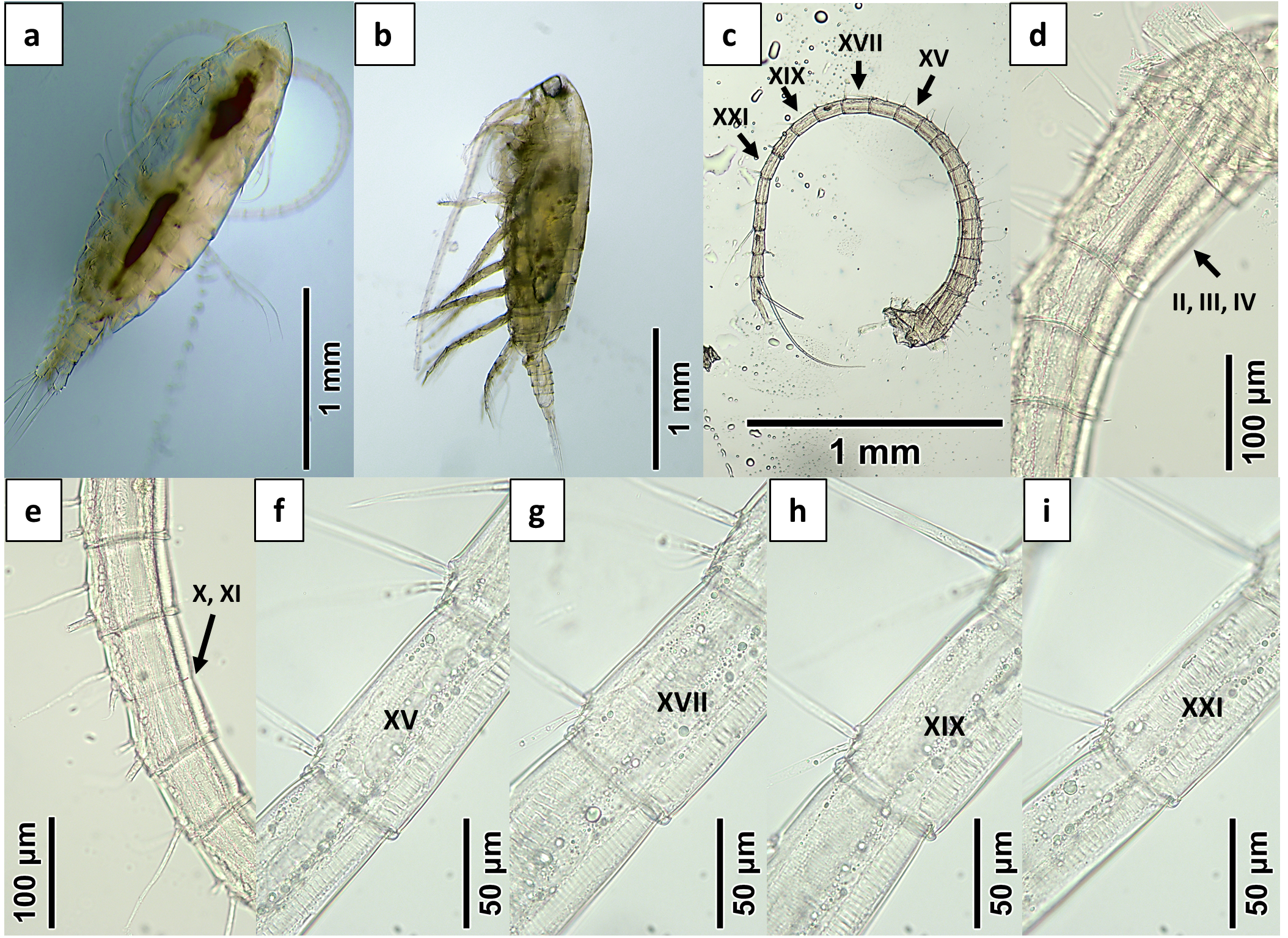
**

**Figure S8:** Photographs of *Calanoides natalis* female specimens from our samples, taken under the microscope: dorsal view (a), lateral view (b), A1 antenna with ancestral segments XV, XVII, XIX and XXI indicated with arrows, containing one seta of modified type and one aesthetasc seta-a morphological feature distinguishing *Calanoides natalis* from *Calanoides carinatus* (c), A1 antenna with ancestral segments II, III and IV partly fused-indicated with arrow (d), A1 antenna with ancestral segments X and XI partly fused-indicated with arrow (d), A1 antenna-ancestral segment XV (f), A1 antenna-ancestral segment XVII (g), A1 antenna-ancestral segment XIX (h), A1 antenna-ancestral segment XV (f).


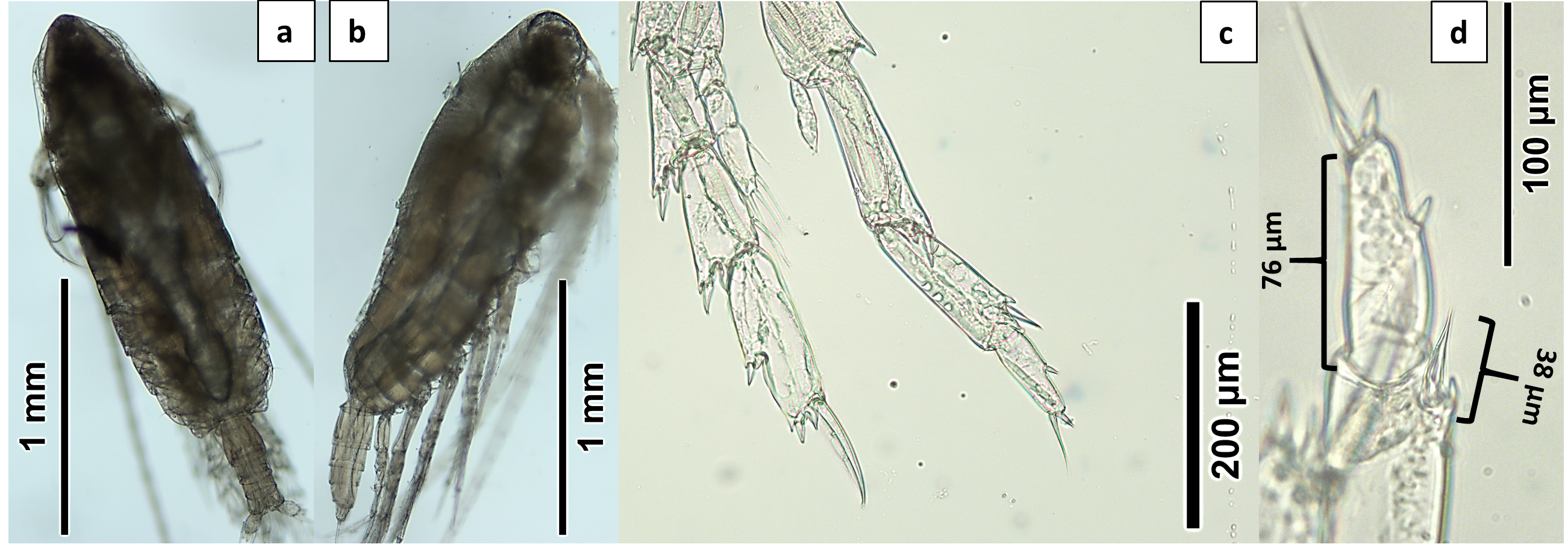


**Figure S9:** Photographs of *Calanoides natalis* male specimens from our samples, taken under the microscope: dorsal view (a), lateral view (b), P5 leg (c), exopodite of P5 leg (d)-the ratio of the length of the outer distal spine of exopodal segment 2 relative to the length of the inner border of exopodal segment 3 is 0.5- for *Calanoides natalis* this ratio has a mean of 0.54 (range 0.47-0.67), whereas in *Calanoides carinatus* this ratio has a mean of 0.96 (Sabatini et al., 2007).

**LITERATURE**

Sabatini, M.E., Ramírez, F.C., Bradford-Grieve, J., 2007. Redescription of *Calanoides carinatus* (Krøyer) (Copepoda: Calanoida: Calanidae) with a discussion on the status of related species. Invertebr. Syst. 21, 341–364.
